# Supplementary material for: Whole-genome ancestry of an Old Kingdom Egyptian
Source: Nature. 2025 Jul 2;644(8077):714–21. doi: 10.1038/s41586-025-09195-5 (PMC12367555; doi:10.1038/s41586-025-09195-5)
Supplement: Supplementary file 1 — Supplementary Information sections 1–5, including figures, tables and references. [file 41586_2025_9195_MOESM1_ESM.pdf]

nature portfolio

https://doi.org/10.1038/s41586-025-09195-5

Supplementary information

Whole-genome ancestry of an Old Kingdom Egyptian

In the format provided by the authors and unedited

SUPPLEMENTARY INFORMATION

WHOLE-GENOME ANCESTRY OF AN OLD KINGDOM EGYPTIAN

Adeline Morez Jacobs[1,2], Joel D. Irish[1], Ashley Cooke[3], Kyriaki Anastasiadou[2], Christopher Barrington[2,4], Alexandre Gilardet[2,5], Monica Kelly[2], Marina Silva[2], Leo Speidel[2,6], Frankie Tait[2], Mia Williams[2], Nicolas Brucato[7], Francois-Xavier Ricaut[7], Caroline Wilkinson[8], Richard Madgwick[9], Emily Holt[9], Alexandra J. Nederbragt[10], Edward Inglis[10], Mateja Hajdinjak[2], Pontus Skoglund[2], Linus Girdland-Flink[1,11]

[1]School of Biological and Environmental Sciences, Liverpool John Moores University, Liverpool, United Kingdom
[2]Ancient Genomics Laboratory, The Francis Crick Institute, London, United Kingdom
[3]World Museum, National Museums Liverpool, Liverpool, United Kingdom
[4]Bioinformatics and Biostatistics, The Francis Crick Institute, London, United Kingdom
[5]Centre for Palaeogenetics, Stockholm 106 91, Sweden
[6]Genetics Institute, University College London, London, United Kingdom
[7]Centre de Recherche sur la Biodiversité et l'Environnement (CRBE), Université de Toulouse, CNRS, IRD, Toulouse INP, Université Toulouse 3 – Paul Sabatier (UT3), Toulouse, France
[8]Face Lab, Liverpool John Moores University, Liverpool, United Kingdom
[9]School of History, Archaeology and Religion, Cardiff University, Cardiff, United Kingdom
[10]School of Earth and Environmental Sciences, Cardiff University, Cardiff, United Kingdom
[11]Department of Archaeology, School of Geosciences, University of Aberdeen, Aberdeen, United Kingdom

Contents

SI 1. Archaeological context 1
SI 2. Osteological analyses of the Nuwayrat individual 12
SI 3. Facial reconstruction of the Nuwayrat individual 43
SI 4. Genetic ancestry modelling and admixture dating 51
SI 5. Stable isotopes analyses 61

SI 1. Archaeological context

*Corresponding authors: Ashley.Cooke@liverpoolmuseums.org.uk (A. C.), linus.girdlandflink@abdn.ac.uk (L. G. M)

The pottery coffin burial was given to World Museum (then Liverpool City Museum) in 1950 by the University of Liverpool from the collections of the old Institute of Archaeology (1904-1948). Following a devastating fire during the Liverpool Blitz of May 1941 the museum was preparing to reopen one part of the building with new displays including the burial customs of ancient Egypt[1]. Egyptologists at the University of Liverpool were assisting curators with a reassessment of the surviving collection, identifying gaps that could be filled from the university's own archaeology collection through loans or donations. In 1936 the Institute of Archaeology had lent to the museum a wooden chest-shaped coffin of the 3rd Dynasty (c. 2686 - 2613 BCE) with a contracted burial. This (and about 3000 other Egyptian and Sudanese antiquities) were destroyed in the fire and so the pottery coffin burial was selected as a near contemporary replacement, accessioned into the museum collection as number 50.33. The accompanying Institute of Archaeology index catalogue card records that the pottery 'archaic coffin', oval-shaped within conical cover containing 'characteristic contracted burial within' was found at 'Beni Hasan' and dates to 'about Dynasty II, about 3000 BC'. The coffin was broken into fragments and would require repair if exhibited; damage that presumably happened when the Institute of Archaeology was hit during the bombing raids of May 1941. Remnants of woven linen textile with selvedge may represent clothing of the deceased placed over the body.

The pottery coffin burial is one of several hundred finds made during the course of excavations carried out in a group of cemeteries near the village of Beni Hasan, c. 265 km south of Cairo (Fig. S1.1), between 1902 and 1904 by an archeological team led by John Garstang of the University of Liverpool[2-6]. Garstang worked on behalf of the Beni Hasan Excavations Committee, a private enterprise body that funded the excavations for two seasons. Between 1884 and 1983 the Egyptian Antiquities Service could grant excavators a division of the finds made each season, reserving the most significant objects for the Egyptian Museum in Cairo[7]. Members of the Beni Hasan Excavations Committee received a share of each season's finds that Garstang was permitted to export. Some committee

members were patrons of the university (such as Sir John Brunner who endowed the first chair of Egyptology) and would donate part of their share to the Institute of Archaeology and in this way the university's collections grew through consecutive fieldwork and the remainder is now housed in the Garstang Museum of Archaeology at the University of Liverpool.

Beni Hasan was well known to Garstang thanks to the existence of a terrace of large brightly decorated tombs cut into the limestone hillside of the east bank of the Nile. They belonged to elite families who governed this region, known as the Sixteenth Upper Egyptian (Oryx) nome, during the Middle Kingdom (11th-12th Dynasties, c. 2055-1773 BCE). Garstang's primary interest was the necropolis there, where his team cleared 888 simple shaft tombs belonging to the families who served the governors of the region[8]. For part of the winter season of 1903-1904 Garstang's team also worked at a lesser-known necropolis 3 km north, in the hillside to the east of a small village called Nuwayrat, excavating about 100 tombs from the early Old Kingdom (3rd-4th Dynasties, c. 2686-2494 BCE) an historical age epitomised by the pyramids of Giza. Garstang describes a mix of small rock-cut tombs in the hillside; and on the escarpment below more simple burials within pottery coffins or beneathinverted pottery vessels, covered over by a circle of rocks. He based his dating of the necropolis on the 'archaic' manner of the burials and the simple construction of the rock-cuttombs[6]. The excavations were published as a single volume in 1907 entitled *The burial customs of ancient Egypt as illustrated by tombs of the Middle Kingdom; being a report of excavations made in the necropolis of Beni Hassan during 1902-3-4*. It was designed more asa textbook on the development of funerary culture in the Middle Kingdom and gives an inadequate five-page report on the 100 tombs excavated in the early Old Kingdom necropolis at Nuwayrat.

With a lack of plans and complete inventories of individual tombs Garstang's field notes fall short of the standards of the time, but he excelled in the use of photography as a medium for documentation. Using a tomb as a darkroom team member Mahmoud Abd El Gelel developed more than 1000 negatives during the two excavation seasons[6]. Working with the glass plate negatives now preserved at the Garstang Museum of Archaeology it is possible to reconstruct the archaeological context of pottery coffin burial 50.33[9]. Garstang divided the necropolis into a southern group of burials dating to the 3rd Dynasty or slightly earlier and a northern group dating to the 3rd- 4th Dynasties[6]. For the latter group only one rock-cut

tomb was found undisturbed and the contents were sent to Liverpool[6]. Garstang describes a small chamber cut into the limestone hillside with, on either side of the entrance, two burial shafts each 1 metre wide and about 1.5 metres deep (Fig. S1.2). The north burial chamber contained the pottery coffin with contracted burial now in World Museum, no. 50.33 (Fig. S1.3). The south burial chamber contained a wooden chest-shaped coffin with a contracted burial, with the limbs wrapped in linen, previously on display in the museum but destroyed by fire in the Second World War (Fig. S1.4).

A new investigation of the Nuwayrat necropolis by the KU Leuven Dayr al-Barsha project in 2006 has largely confirmed Garstang's dating for the necropolis[10]. Pottery from burial goods (or offerings for the dead) found outside the rock-cut tombs and around the rock circle burials was dated to the 3rd Dynasty to early 4th Dynasty. The size of the necropolis is far larger than Garstang reported, with field surveys by Bart Vanthuyne recording hundreds of rock circle tombs spread across 2.6 km of the escarpment[11,12]. Vanthuyne's research of the earliest rock-cut tombs in provincial Egypt included permission from World Museum in 2016 to C14 radiocarbon measure a sample of skeleton 50.33. Michael Dee at the Oxford Radiocarbon Accelerator Unit, University of Oxford, obtained a date of 4093 ±35 BP (ref. OxA-33186) that calibrates to a –2870-2490 cal BCE (95.4% probability), a date range that straddles the 3rd - early 4th Dynasties.

We merged OxA-33186 with two new dates (Beta – 635236 and Beta - 635237) obtained from tooth collagen of two teeth, one of which we generated the majority of genome data from (Supplementary Data Table S2). The combined date (see Methods) yielded a conventional radiocarbon age of 4098 ± 19 BP, which calibrates to 2855-2570 cal BCE (95.4% probability). However, the conventional radiocarbon age intersects with the calibration curve over two so-called wiggles and a weak plateau (i.e. natural variations in atmospheric C14) which results in trimodal probability distribution (Fig S1.5). This means that the maximum intercept range (at 95.4% probability) is conservative.

We attempted to resolve these issues by leveraging the fact that dentine forms over a limited period and does not turn over later in life, meaning that no new collagen is synthesised in those tissues once fully formed. Therefore, C14 measurements on collagen from dentine represent an average of the period of tissue formation. Conversely, femur collagen is gradually replaced ('turned over'), meaning that a certain proportion of older collagen is

continuously replaced with newly synthesised collagen. The collagen in a femur of a 45-60-year-old male should therefore represent an average formation age that is closer to the age of death than the M3 collagen. This age-gap can then be implemented in Bayesian modelling during C14 calibration. The average formation age for M3 collagen is approximately 20 years of age[13,14] .

However, upon closer review of the collagen turn-over rates in femurs, we find that adult femurs will always contain a substantial proportion of collagen synthesised prior to the age of 20-25[15] such that an age-weighted average in adult femur collagen will always be markedly younger than the age-at-death. Specifically, Hedges et al. (2007)[15] estimated that collagen turn-over rates between the ages of ~10-20 are much higher (~15-25% for the best-fit model) than from ages ~25-80, during which it gradually decreases from ~3-1.5%. Moreover, they also observed up to 30% individual variation in turn-over rates. Therefore, the precise amount of pre-age 20 collagen that is retained at any given age in adulthood is difficult to determine with precision; it is also not known whether there is variability in turn-over rates for age-specific fractions in the total collagen pool. Consequently, since adult femur collagen will always contain a substantial proportion of collagen formed in adolescence, and/or before the age of 20, it is not suitable for Bayesian gap modelling; in fact, it is likely that part of the collagen from which the C14 date was derived formed at an earlier age (i.e. is older in C14 years) than the collagen from the M3 tooth.

The early Old Kingdom was a time of change for people living in the provinces of Egypt such as the Oryx nome in Upper Egypt where modern Nuwayrat lies. Central administration became more visible to rural communities through the establishment of royal domains that sought greater control of resources. Administrators worked to secure provisions for royal mortuary cults, temples maintained by the state and construction projects, particularly pyramid building[16]. The shape of royal architectural programmes changed dramatically in the Third Dynasty when king Netjerikhet (better known as Djoser) built the Step Pyramid at Saqqara (c. 30 km south of Cairo), the oldest of all Egyptian pyramids. About 80 years later King Khufu of the Fourth Dynasty built the largest pyramid at Giza, part of the same necropolis as Saqqara that served the royal residence at Memphis. The tombs of high officials buried around the king included underground burial chambers exclusively for the dead and their burial goods, with an aboveground chapel where the living could make offerings.

The shape of coffins also changed at the start of the Old Kingdom, with royalty and the elite moving away from contracted burials, favouring the body lying stretched out and flat, something most likely connected with innovations in embalming at the time[17]. Ancient Egypt was a class society and these changes at the royal residence would slowly began to influence burial customs for some elite people in the provinces. Even in the early age of the pyramids, contracted burials within a ceramic coffin or vessel remained a common funerary practice in provincial cemeteries[10,18,19]. However, at Nuwayrat we can observe variation in the necropolis of the nearby rural population living in small settlements spread along the Nile valley. The rock-cut tombs hewn out of the cliffs required far greater resources to construct than the rock circle tombs in the escarpment. With their subterranean burial chambers and superstructures (perhaps used as a chapel for visitors), they resemble to some extent the funerary architecture seen in cemeteries of the royal residence. The people buried in these rock-cut tombs (such as World Museum 50.33) appear to be of a higher social class than the majority of others in the necropolis. Given theincreasing presence of royal authority in the countryside these people may belong to the elite of a royal domain or have connections with state administrators and a familiarity with funerary practices in the royal cemeteries[10].

References

[1]Allan, D. The Destruction of the Liverpool City Museums: a Review of Events. Museums Journal 41. **5**, 105-106 (1941).

[2]Garstang, J. Excavations at Beni-Hasan, 1902–3. *Man: A Monthly Record of Anthropological Science.* **3**, 97–98 (1903a).

[3]Garstang, J. Excavations at Beni-Hasan, 1902–3 (ii). *Man: A Monthly Record of Anthropological Science*. **3**, 129–130 (1903a).

[4]Garstang, J. Excavations at Beni-Hasan in Upper Egypt (Second Season). *Man: A Monthly Record of Anthropological Science*. **4**, 97–99 (1904a).

[5]Garstang, J. Excavations at Beni Hasan (1902–1903–1904). Annales du Service des Antiquités de *l'Égypte*. **5**, 215–228 (1904b).

[6]Garstang, J. 1907. *Burial Customs of Ancient Egypt: as Illustrated by Tombs of the Middle Kingdom being a Report of Excavations Made in the Necropolis of Beni Hassan During 1902–3–4.*(London: Constable, 1907).

[7]Stevenson, A. *Scattered Finds: Archaeology, Egyptology and Museums*. (London: UCL

Press,2019).
[8]Snape, S. *Ancient Egyptian Tombs: the Culture of Life and Death.* (Malden, MA; Chichester:Wiley-Blackwell, 2011).
[9]Vanthuyne, B., Dee, M., Dupras, T. McGinn-Roberts, C., Cooke A., & Criscenzo-Laycock, G. Early Old Kingdom Tombs at Nuwayrat and Zawyet Sultan, 7th Conference of Old Kingdom Art and Archaeology conference. Università degli Studi di Milano, May 8-12, 2017 (2017).
[10]De Meyer, M., Stefanie V., Vanthuyne, B., Hendrickx, S., Op de Beeck, A., & Willems, H. The Early Old Kingdom at Nuwayrāt in the 16th Upper Egyptian Nome, in: Aston, David, Bettina Bader, *Carla Gallorini, Paul Nicholson and Sarah Buckingham (eds.). Under the Potter's Tree: Studies on Ancient Egypt Presented to Janine Bourriau on the Occasion of her 70th Birthday*.Orientalia Lovaniensia Analecta 204, 679–702. (Leuven: Peeters, 2011).
[11]Vanthuyne, B. The Beni Hasan el-Shuruq region in the Old Kingdom: A preliminary survey report. *Prague Egyptological Studies.* **21**, 94-105 (2018a).
[12]Vanthuyne, B. Petrie in Nuwayrat. *Egyptian Archaeology.* **53**, 44–46 (2018b).
[13]Berkovitz, B. K. B (ed). Why Should It Matter How Long Our Ancestors' Teeth Took To Develop? in *Nothing but the Tooth*. 175-188. (Elsevier: 2013)
[14]Putul, M., Konwar, R., Dutta, M., Basumatary, B., Rajbongshi, M. C., Thakuria, K. D., Sarma, B. Assessment of Age at the Stages of the Eruption of Third Molar Teeth among the People of North-Eastern India. *Biomed Research International.* **1**, 9714121 (2021).
[15]Hedges, R. E., Clement, J. G., Thomas, C. D., O'connell, T. C. Collagen turnover in the adult femoral mid-shaft: modeled from anthropogenic radiocarbon tracer measurements. *American Journal of Physical Anthropology*. **133**(2):808-16 (2007).
[16]Guyot, F., Azzarà, V., Briois, F., Marchand, J., & Midant-Reynes, B. An Early Old Kingdom Rural Community in the Eastern Nile Delta. *Bulletin de l'Institut français d'archéologie orientale*. **118**, 143-187 (2019).
[17]Cooney, K. Coffins, Cartonnage, and Sarcophagi in Melinda Hartwig (ed.) *A Companion to Ancient Egyptian Art*. (Boston MA and Oxford: Wiley-Blackwell, 2015).
[18]Grajetzki, W. *Burial Customs in Ancient Egypt, Life in Death for Rich and Poor.* (London: Duckworth, 2003).
[19]Cotelle-Michel, L. *Les sarcophages en terre culte en Egypte et en Nubie: de l'époque prédynastique à l'époque romaine*. (Dijon: Faton, 2004).

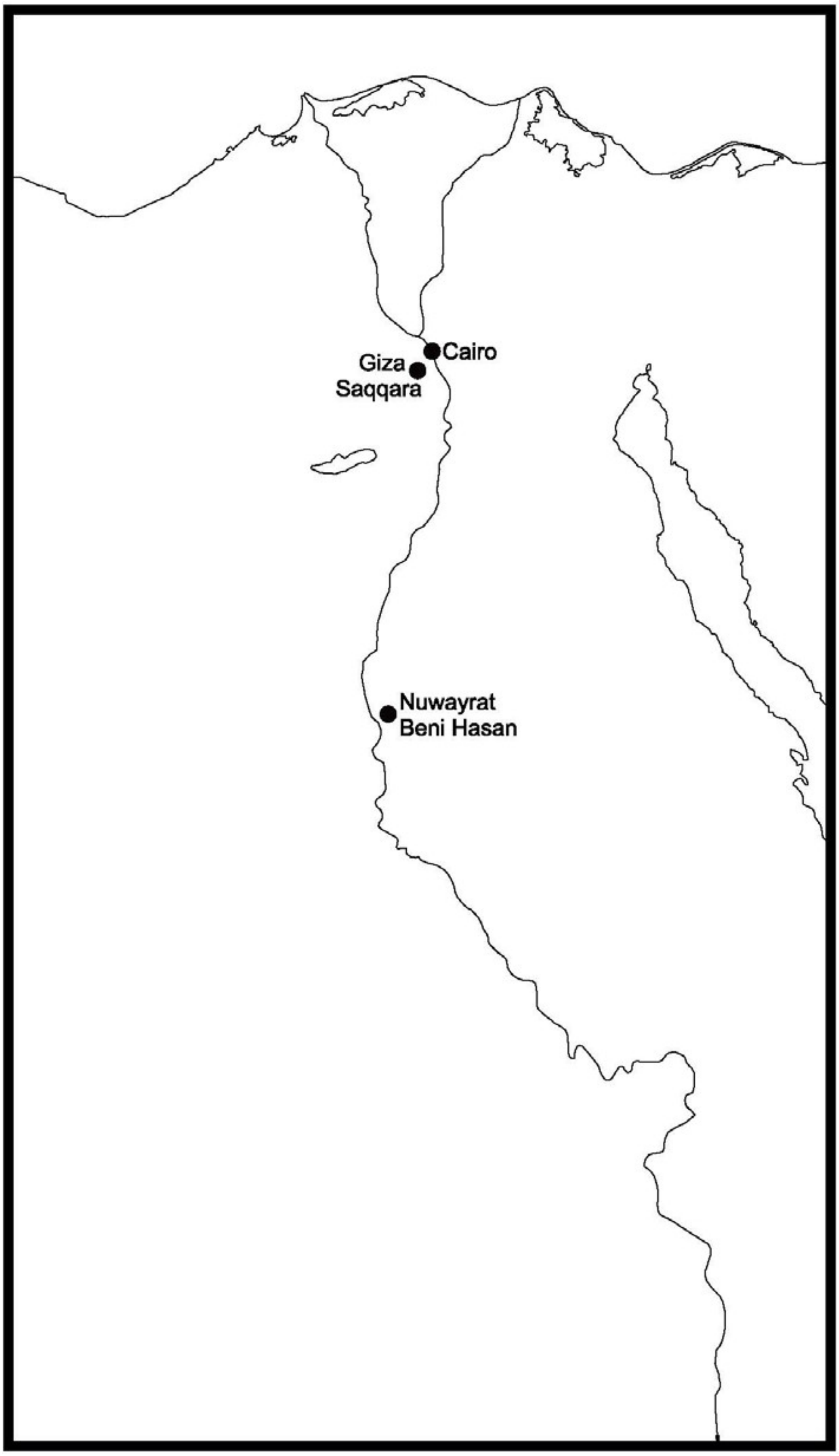

Figure S1.1. Map showing the location of Beni Hasan & Nuwayrat (adapted by A. Cooke from © OpenStreetMap)

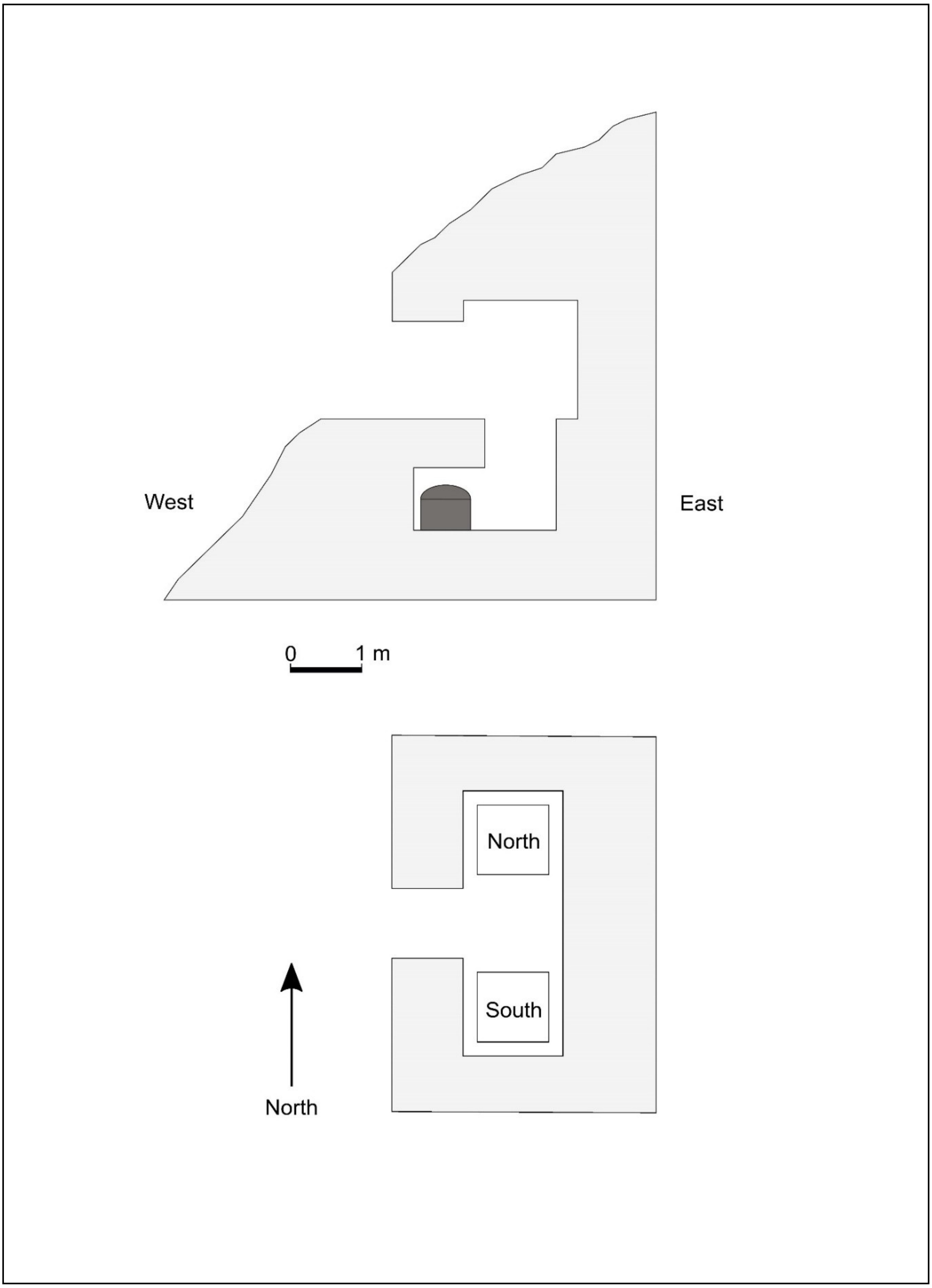

Figure S1.2. An impression of the rock-cut tomb (A. Cooke).

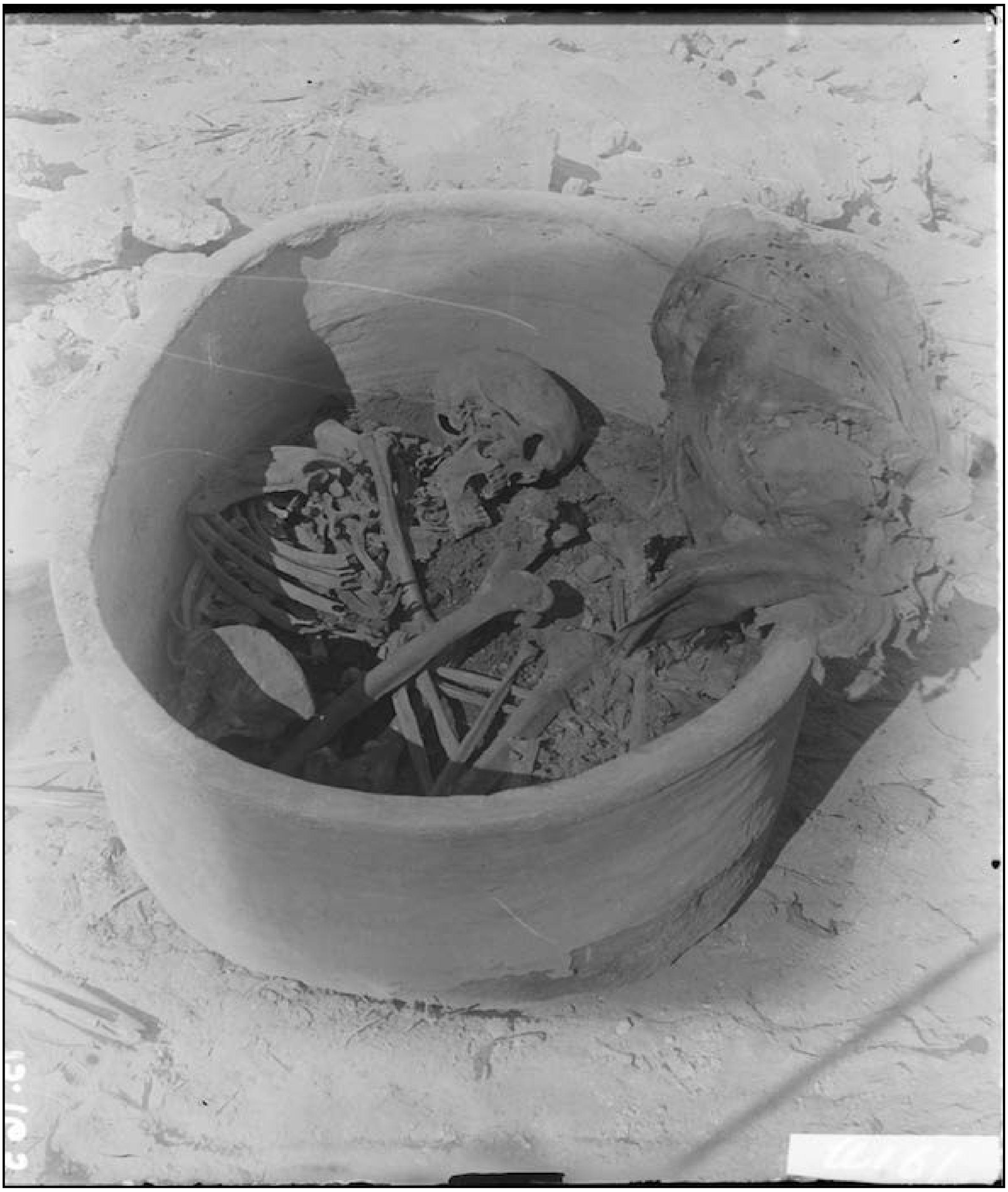

Figure S1.3. Excavation archive image of a contacted burial within a pottery coffin, excavated at Nuwayrat 1903-1904, and thought to be World Museum 50.33 (image courtesy of the Garstang Museum of Archaeology, University of Liverpool).

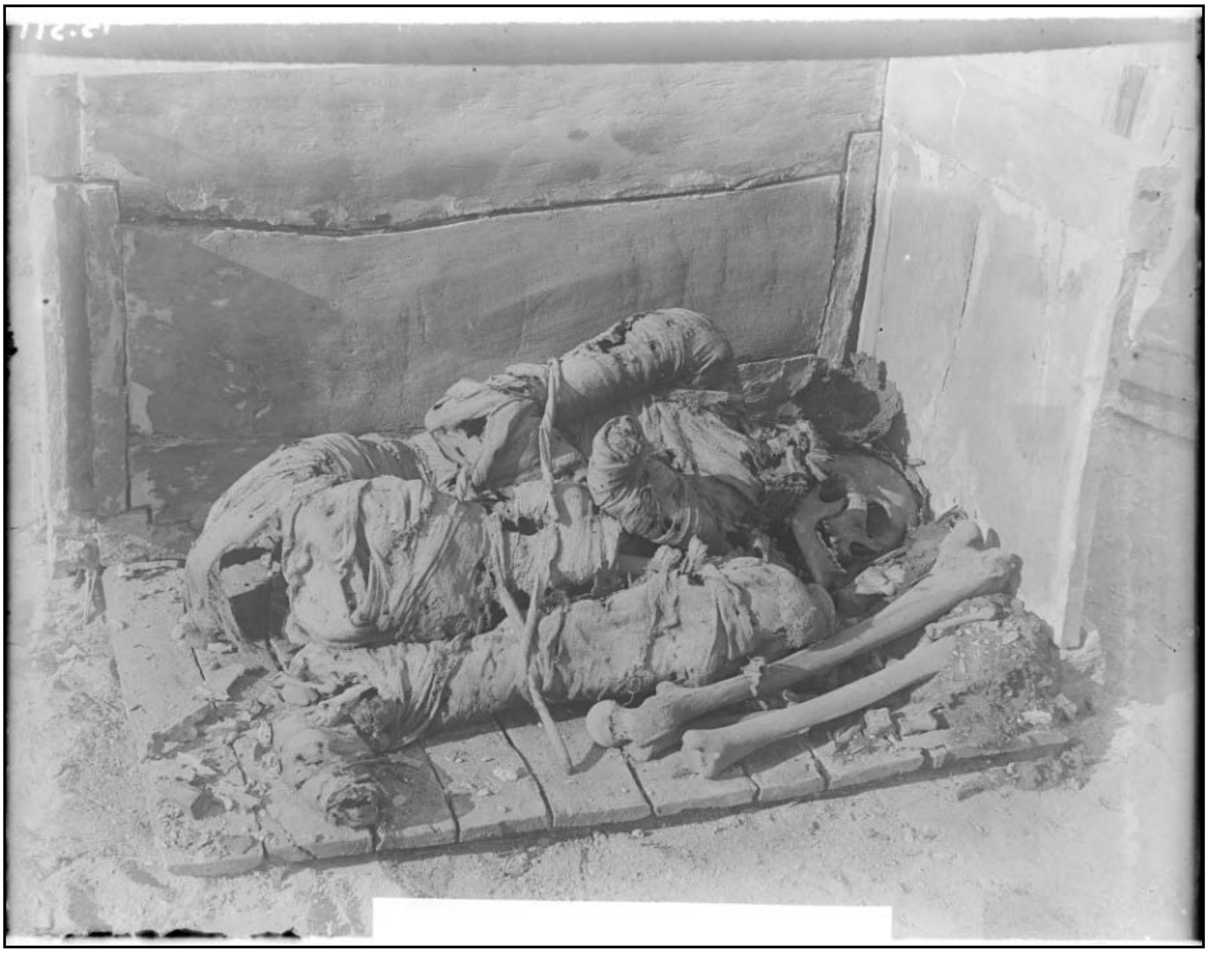

Figure S1.4. Excavation archive image of a contracted burial within a wooden coffin, excavated at Nuwayrat 1903-1904, destroyed in the Second World War when on display within World Museum (image courtesy of the Garstang Museum of Archaeology, University of Liverpool).

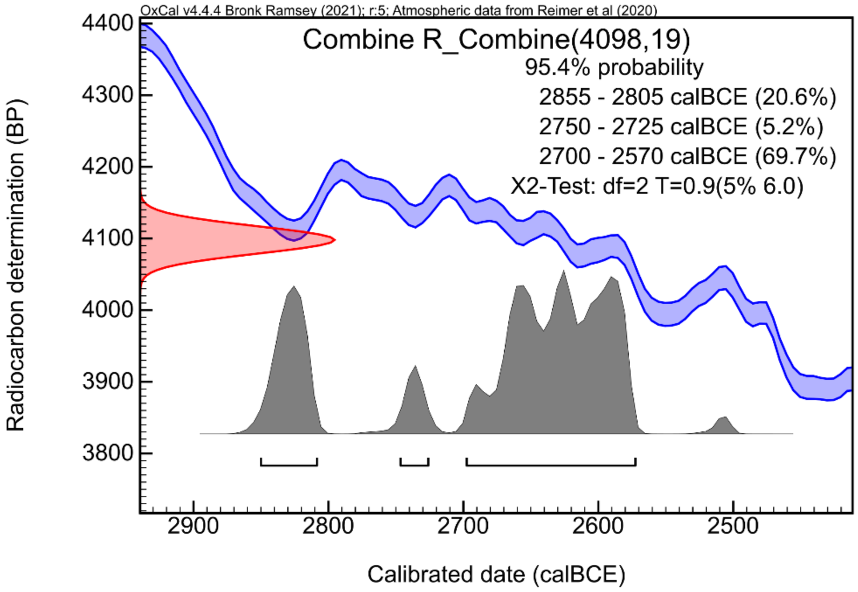

Figure S1.5. Calibrated curve for the combined ^{14}C radiocarbon dates from the Nuwayrat individual. The red curve presents the uncalibrated probability distribution of the three combined C14 dates. The grey curve represent the calibrated density distribution of the combined C14 dates (95.4% probability distribution represented with the bars). The blue curve represents the calibration curve ± 1 s.d.

SI 2. Osteological analyses of the Nuwayrat individual

*Corresponding author: J.D.Irish@ljmu.ac.uk (J.D.I.)

Inventory

Although apparently whole upon its initial recovery, the well-preserved ancient DNA (aDNA) donor skeleton is now <90% complete (Fig. S2.1). It lacks a few small cranial bones, i.e., the left and right lacrimals, vomer, left and right nasal conchae, hyoid, and several maxillary teeth (see below), along with a greater number of missing post-cranial elements; these include the: right clavicle, manubrium, seven vertebrae—C1, C2, T7, T9, T11-12, and L4, right radius and ulna, several ribs—primarily 7-11, left patella, left fibula, most bones of both hands and, to a lesser extent, the feet. The third and fourth metacarpals (MC) and two proximal phalanges of the left hand remain. The calcaneus, talus, and fifth metatarsal (MT) of the left foot are present, as are these elements along with the first and second cuneiforms, MT4, and proximal hallux phalange of the right foot. In several cases, the extant postcranial bones are incomplete or otherwise unobservable due to damage including, most notably, the left proximal humerus, left ulna head, proximal and distal ends of most ribs, C5-6 vertebral arches, crests of the right and left ilia, and the superior symphyseal surfaces of both pubic bones, among others.

Biological sex

The genetic results in the main text reveal that the individual was male. This determination is supported by a range of standard nonmetric skeletal indicators[1]. Beginning with the skull, the: supraorbital ridges are pronounced with a concomitant retreating forehead, superior orbit borders are blunt, external occipital protuberance is developed, mastoid processes are large, suprameatal crests are present, mental eminence is square, and gonial angles are near 90 degrees (Fig. S2.2). Among other indicators in the post-crania, the: sciatic notches of both ilia are narrow, ventral arcs are lacking, sub-pubic concavities are absent, superior inlet is heart-shaped, incomplete auricular surfaces appear flat, pre-auricular sulci are absent, and sacral body is curved with body height greater than that of the ala. The only postcranial indicators at odds with a determination of male are metric, i.e., small humeral and

femoral head diameters, which are simply consistent with the overall small size of the individual (below).

Age

Full closure of all epiphyses, among other skeletal evidence (e.g., third molar eruption, tooth wear/loss, etc.), indicate the individual was an adult at the time of death. However, several methods can more precisely estimate his age. The first used here, i.e., based on appearance of the fourth rib sternal end[2-4], has received criticism[5-6], including an inability to correctly identify the requisite rib in cases of damaged or missing elements (above). Nevertheless, two putative fourth ribs (with scores of M8A and M8C according to the method) suggest an age of >64 years. The second method is cranial suture closure[7], which is also considered to be less precise than some. But near and complete fusion of nine of 10 ectocranial sutures (Fig. S2.2) implies ages of 51.5+/-12.6 (with the sum of scores for sutures numbered 1 to 7 equalling 20) and 56.2+/-8.5 years (ribs 6 to 10=14)[see 7]. Third, aging based on the pubic symphyses is a more accepted approach, with several scoring systems available. However, caution is warranted given the damaged superior symphyseal surfaces. According to Todd's[8] method, here based on the extant inferior surfaces, bilateral scores of IX indicate an age of 44-50 years. With the Suchey-Brooks method[1,9], right and left scores of V-2 correspond to 45.6+/-10.4 years with a range of 27-66. Finally, the two auricular surfaces—though also damaged—were scored as 8[see 10], for an age of 60+ years. In summary the age range is ~44- 64 years, though ossification of the costal cartilage, general osteoarthritis—some severe, lack of bone density (osteoporosis), and advanced tooth crown wear (below) suggest an age at death nearer the upper end of this range.

Stature

The long-used regression formulae of Trotter and Gleser[11], based on long bone dimensions in American White and American Black males[tables in 12], returned a wide range of 156-168 cm for the individual (Table S2.1). The mean White estimate is 163.37 cm—after the subtraction of a 0.06 correction factor for individuals over 30 years of age at death[12]. The corresponding mean from Black males is >4 cm shorter, at 159.23 cm. Of these, it was suggested that the Black estimates are more

representative of ancient Egyptians[13], the identified ancestry of the present individual notwithstanding (also below). More recently Raxter and colleagues[14], based on stature reconstruction of ancient Egyptian skeletons and, again, modern American Whites and Blacks, calculated a new set of formulae (Table S2.1) in a combination of long bones. The results correspond overall with Black stature estimates but provide a tighter cluster of results that these authors maintain provide the best ancient Egyptian statures. The range for the Nuwayrat individual is 157.37-160.52, with a mean of 159.06 cm following the 0.06 correction (i.e., 62.62 in; 5 ft., 2¾ in).

Biological affinity

The genetic ancestry of the individual has been determined. However, many nonmetric and metric skeletal based methods have long been used to estimate biological affinity, under the assumption that they are genetically determined[12,15,16]. Often used in forensic contexts, all have come under increased scrutiny for reasons ranging from methodological to ethical.

They can be particularly problematic in individuals of admixed ancestry or, as in the present study, archaeological specimens[overview in 16]. That said, two recognized methods, while not free of such criticism, are used here: 1) dental nonmetric traits, using a program incorporating the Arizona State University Dental Anthropology System, or ASUDAS[17,18], and 2) craniometrics[19]. In the process, because the ancestry of the Nuwayrat individual is known the validity of these methods can be evaluated, at least in this specific case.

Dental traits from the ASUDAS were shown to be reliable proxies for DNA on continental and global levels when comparing samples[20]. This reliability is diminished at an individual level, but a method is available. Denoted as rASUDAS (https://osteomics.com/rASUDAS2/), an unknown individual can be compared with seven global samples based on the presence/absence of up to 32 crown and root traits; a naive Bayes classification algorithm calculates the probability of group membership[21]. Many Nuwayrat teeth (see Fig. S2.3) have incomplete crowns, are heavily worn, or were lost post- and ante mortem (i.e., left UI1-UI2, UM1, UM3 and right UP1, UM2-UM3). Thus, only 15 ASUDAS traits could be scored (Table S2.2). On this very limited basis the individual was compared with only the two most

geographically proximate samples in the program: Western Eurasia and sub-Saharan Africa. The posterior probabilities of belonging to the former is 0.99, while the latter sample is 0.01.

Twenty-nine craniometric measurements (Table S2.3) from Howells[19] were taken of the Nuwayrat cranium for the CRANID program CR6bIND[22]. CRANID compares an unknown individual with 74 premodern through recent global samples. Results from the linear discriminant function analysis in the program revealed that the Nuwayrat cranium most closely resembles, in descending order: 1) a sample of Iron Age male crania from West Asia (i.e., Middle East), namely Lachish, Palestine, with a probability of group membership at 0.530, 2) the female crania from that site, 0.254, and 3) an Egyptian 26th-30th Dynasty sample of males, at 0.175. From this, group probabilities drop off precipitously at 0.02 through 0.00 for the remaining 71 samples. It should be noted that relative to the genetic findings in the main text, neither Neolithic North African nor other specific West Asian cranial samples are part of the CRANID database for direct methodological comparison. So the present craniometric classification are based only on those samples which are included and of greatest similarity.

Next, according to the CRANID nearest neighbour discriminant analysis, the individual cranium most like Nuwayrat is from a West Asian Bedouin male (Individual 2546 in CRANID database), with the following rounding out the top five: Egyptian 26th-30th Dynasty male (Ind 1034), Indian male (2576), Lachish male (2668), and another 26th-30th Dynasty Egyptian male (1031).

Thus, in line with the genetic results the Nuwayrat individual, subject to limitations imposed by the comparative samples available in the two program datasets (as above), appears most akin phenetically to: Western Eurasians rather than sub-Saharan Africans dentally and, more specifically, premodern West Asians, i.e., Lachish, based on craniometrics. It is secondarily most similar in craniometric dimensions to ancient Egyptians of a more recent time.

Paleopathology

There are no signs of acute trauma, congenital anomalies, infectious disease,

haemopoietic disorders, metabolic disorders, tumours, or other overt condition in the skull or post- crania[1,23-24]. Two exceptions, as mentioned, are: 1) slight osteoporosis, indicated by relatively light weight, thin cortex, and a loss of (visible) trabeculae in most elements, and 2) osteoarthritis (OA) of multiple joints (a.k.a. degenerative joint disease, or DJD) and the vertebral bodies (degenerative disc disease, or DDD). Of interest, OA, as manifested by osteophytosis (lipping), porosity, and/or eburnation (polishing), varies markedly throughout the skeleton. Many joint surfaces suggest basic age-related DJD, including the: 1) shoulders (glenoid fossa, acromion, clavicle, humeral head), 2) elbows (distal humerus, proximal left ulna and radius), 3) left wrist (distal radius), 4) pelvic girdle (auricular surface, pubic symphysis, acetabulum, femoral head), 5) knees (distal femur, proximal tibia, patella), and 6) ankles (distal tibia and fibula, talus, calcaneus). Joint surfaces of the left MC3 and MC4, two more right tarsals, and the left MT5 are unremarkable as well in appearance. Regarding DDD, the thoracic and lumbar vertebrae show what may be expected as normal age-related wear, with possible exception (below).

In contrast, atypically severe DJD is present in the form of: 1) porosities, osteophytosis, and slight eburnation of the right temporomandibular joint (TMJ) (Fig. S2.3a), 2) eburnation and osteophytosis of facets between the right MT4 and MT5 (below), contra the left MT5, and 3) considerable eburnation and osteophytosis on the distal joint of the proximal phalange of the right hallux (below). The latter is indicative of chronic dorsiflexion[25]. The five surviving cervical vertebrae show extensive pitting, osteophytosis, and some eburnation on the articular facets (DJD)—particularly C3 and C4 (Fig. S2.4a), and bodies (DDD)—most notably C5-6, which are fused, and C7 (Fig. S2.4b). Such wear can result from repetitive flexion, i.e., looking down. The extant thoracic and lumbar vertebrae are affected to a lesser degree, though the T6, T8, L3, and L5 bodies have considerable anterior osteophyte development. This patterning, together with entheseal changes at certain muscle origin and insertion sites, indicates repetitive movements exceeding normal joint lever action; they may be indicative of the individual's life history, as detailed below.

Finally, focusing on the teeth, the remnant crowns are worn (see Fig. S2.3), which is common in older adults and not considered pathological[26]. However, the molars are extremely worn. The anterior teeth evidence attrition resulting from normal

mastication, having wear grades of ~2 (dentine exposure) in the ASUDAS[17,18]. The molars were affected by attrition, but also by evident abrasion on the right teeth showing extreme buccal wear relative to the left. Abrasion occurs from contact with foreign objects, e.g., using the teeth as tools[26]. Molar wear grades range from 3 (remnant enamel ring) to 4 (crown worn away completely) in the maxillary right first molar. The latter wear penetrated the pulp cavity (Fig. S2.3b), which became infected to produce a large unhealed alveolar abscess. The strongly angled wear of the right molars, requiring a substantial bite force, is likely linked with the abovementioned TMJ damage. Together, this is suggestive that the individual used these teeth like a vise/pliers or, less likely, due to the bite force and far-posterior location in the dentition, for stripping fibrous material. Beyond that, except for some alveolar resorption (periodontitis), the dentition was healthy, with no evidence of caries or other intraoral pathology.

Skeletal markers of physical stress

It has long been held that OA location and severity, together with activity-induced musculoskeletal stress (MSM), among various additional markers (e.g., enlargement of ligament attachments and articular margins) can provide insight into an individual's life history including putative occupation(s)[25,27-28]. Such studies remain common[29-32], though recently the concept has been questioned. That is, beyond being entirely circumstantial, such evidence is said to be only marginally informative, if not largely age related[33-34].

Nevertheless, the Nuwayrat skeleton has unique DJD and DDD patterning, plus other conspicuous changes including localized muscle attachment robusticity in an otherwise gracile skeleton. Thus, with the potential caveat of over- or even misinterpretation, these markers, the motions likely responsible for formation and, ultimately, the possible cause(s) are presented. In addition, these markers were quantified based on standard methods[1,28] (as detailed below).

Beginning with the skull, two markers stand out. First, DJD of the TMJ is likely related to gripping or, less likely, stripping some type of foreign object or material in the right molars (again Fig. S2.3). Second, the external occipital protuberance (EOP) is unusually well-developed (Fig. S2.2b), even for a male. In life the nuchal

ligament runs between it and the C7 spinous process, attaching the upper trapezius muscle to the C2-C6 processes. Hyper- development of the EOP is attributed to chronic loading from the weight of the head tilting forward[35-36].

In the upper half of the post-cranial skeleton, the glenoid fossae of both scapulae exhibit moderate marginal osteophytosis, which may simply be age-related but result from arm extension[27]. More unique markers are bilateral supraglenoid articular facets (Fig. S2.5a), which occur from repeated raising of the arms above shoulder height[25]. Bilateral thickening of the lateral scapula margins, from expanded origin points for the teres major and minor muscles (Fig. S2.5b), is discernible with equivalent raised insertions on the humeri (below); this evidences the raising of both arms to shoulder height. Even more pronounced on these margins are the origins for the long head of the triceps brachii, used to extend the elbows. The origins for the deltoid muscles, including the dorsal spines and lateral acromion processes of both scapulae, are well developed to match pronounced deltoid tuberosities of the humeri (Fig. S2.6a). These muscles abduct, flex, and extend the humeri. Also evident on the latter are large insertions for the teres major (above), pectoralis major, and latissimus dorsi muscles. Beyond flexion and extension, these MSMs point to habitual abduction, adduction, and movement of the arms across the chest[25]. In the bones of the extant left forearm the only overt marker is extensive breakdown of the radial tuberosity on the radius (Fig. S2.6b). As the insertion for the biceps brachii, this is indicative of chronic heavy loading of the extended forearms with, unlike above, the humeri positioned down[25]. Finally, the severe cervical vertebrae DJD and DDD indicate chronic neck flexion, with the thoracic and lumbar anterior osteophytes perhaps implying same for the trunk, as in habitually leaning forward.

In the lower half of the skeleton both ischia manifest roughened expanded dorsal surfaces (Fig. S2.7a). They are origin sites for several muscles comprising the hamstrings, which flex the knees and extend the legs (see below). However, the associated insertions on the posterior distal femora and proximal tibiae and fibulae are not enhanced. Thus, the aetiology is more likely related to chronic inflammation of the soft tissue and bursitis to yield ischial osteitis. The left ischium is most affected. This condition can result from sitting for long periods on a hard surface[25,27]. The

remainder of the pelvis is unremarkable in appearance, although as mentioned it is damaged so the state of several key muscle origins cannot be assessed. The proximal femora exhibit multiple stress markers. First, the fovea capitis in both, which function to connect the bone to the acetabulum via the ligamentum teres, are enlarged in diameter and depth (Fig. S2.7b). Chronic stretching of this ligament is indicated. Second, exostoses in both trochanteric fossae, insertions for the obturator externus, are attributed to sitting for long periods with legs flexed straight out[25,37]. Third, in opposition, the gluteal tuberosities for the gluteus maximus attachment are strongly developed from bringing both legs back, i.e., extension. Fourth, the two greater trochanters, where the gluteus medius and minimus muscles insert, are enlarged (Fig. S2.8a); these muscles spread the legs apart, i.e., abduction. Again, showing antagonistic movement, the substantially raised linea apsera on both femora indicates adduction with the adductor longus muscle, i.e., to bring the legs together[25,27]. Finally, though the knees appear largely unaffected, matching facets on the distal anterior tibiae and superior heads of the tali imply habitual squatting (Fig. S2.8b) or kneeling with the feet dorsiflexed. The latter is suggested by the severe DJD of the hallux proximal phalange (Fig. S2.9a)[25,27]. Eburnation between right MT4 and MT5 (above) is unusual in a joint of such limited movement (Fig. S2.9b). It is even more unique in that the same facet on the left MT5 shows no DJD, to suggest differences in how the two feet were used over time.

Together, they all suggest that the individual commonly sat on a hard surface, on or near the ground given his alternate but habitual positioning of his legs straight out and spread vs. drawn-in to squat and/or kneel. In the upper body he habitually held his arms straight out front, but also moved them to the sides and across the chest, at or above shoulder height. Yet he also may have carried heavy loads in front of him with lower arms flexed at the elbows. It is further suggested he leaned forward a great deal of the time, particularly at the neck as in looking downward. And he gripped some sort of non-food item(s) in his back right teeth as if using them like a vise or, less likely, to strip material. The severity scores and grading system used here are provided in Tables S2.4-S2.6. The codes in each table apply to Figure S2.10, which illustrates the location of all markers throughout the Nuwayrat skeleton.

Based on these repetitive, seemingly diagnostic movements, a review of ancient Egyptian tomb and other illustrations was conducted to identify potential occupation—again with the abovementioned caveats in mind. The most commonly depicted occupations requiring physical activity include baker, farmer, mason, soldier, and weavers, among others. Though a few tasks cannot be ruled out (e.g., weaver), it appears that the closest fit is a potter. For example, in the 5th Dynasty (c. 2450-2300 BCE) tomb of Ty, an elite member of society from Saqqara, Egypt, activity in a potter's workshop is present, as illustrated in Plate LXXI from a 1939 volume by Épron and Daumas[38]; to paraphrase a discussion of this illustration by Doherty[39], it shows a range of positions by workers, including: 1) sitting on the ground with legs drawn in facing the kiln, 2) sitting with one arm extended to form a pot while the other arm turns a pottery wheel, 3) standing while holding a large pot with elbows bent, and 4) others performing allied tasks. The pottery wheel was likely introduced by the 4th Dynasty (c. 2600 BCE) from West Asia. Prior to that the manufacturing process was identical, only with pottery instead made via coiling on the ground[39]. Similar body positions are seen in wall decorations from the 12th Dynasty (c. 1938-1773 BCE) tomb of Amenemhat I at Beni Hasan, just three km south of Nuwayrat[40]; in Figure S2.11a, potters are shown working on a bowl, stoking a kiln, carrying a pot, turning a wheel, working at the kiln, carrying multiple vessels, working the clay and, again using a wheel[39]. Finally, in a tomb painting from Thebes in the 18th Dynasty (1427-1401 BCE), again following Doherty[39], from left to right in Figure S2.11b[41] are: 1) a seated potter, leaning forward with arms and legs in front to form a large vessel, with his right foot to steady the wheel, 2) his assistant leaning forward while kneeling with feet dorsiflexed to turn the wheel, 3) a third mixing clay with his feet, and 4) a fourth individual with arms raised tending the kiln.

Many other depictions of pottery making, including statuettes and small wood models, show similar scenes across both time and space in Egypt, with techniques differing little [overview and multiple sources in Doherty[39]; also see image of the latter author (in Figure 6.17 of that thesis) seated on the ground forming a pot on a reconstructed Egyptian wheel with her arms and legs outstretched]. Further, while again circumstantial, the Nuwayrat individual's implied occupation may supported by his interment in a ceramic vessel, though such a practice was not entirely unique during

this period, including at Nuwayrat[42]. A potential cause for the TMJ and molar wear is not apparent in any Egyptian imagery.

Summary

Diagnostic morphological features of the well preserved, <90% complete skeleton from Nuwayrat, with catalogue number 50•33, indicate that the individual was a male, in agreement with the genetic data, perhaps ~60 years of age. He was relatively short, estimated at just over 159 cm, and had a gracile build. Also in agreement with the genetic findings, he has dental similarities to a comparative sample of Western Eurasians and, more specifically, a West Asian sample from Lachish based on craniometric dimensions. His skeletal condition appears consistent with that of an older adult, with osteoporosis and general osteoarthritis (OA), although with more localized and severe OA evident. The only overt pathology is a large alveolar abscess from pulp perforation of the maxillary right first molar. Lastly, though entirely circumstantial, among other concerns with interpretations of this kind, localized OA (with TMJ exception noted), with the appearance of related muscle attachments and additional skeletal stress markers are consistent with the movements and positions of a potter, as indicated in ancient Egyptian imagery.

References

[1]Buikstra, J.E. & Ubelaker, D.H. (Editors). *Standards for Data Collection from Human Skeletal Remains*. (Fayetteville: Arkansas Archaeological Survey, 1994).

[2]Işcan, M.Y. & Loth, S.R. Determination of age from the sternal rib in white males: a test of the phase method. *Journal of Forensic Science*, 31(1), 122-132 (1986).

[3]Işcan, M.Y., Loth, S.R. & Wright, R.K. Age estimation from the rib by phase analysis: white females. *Journal of Forensic Science,* 30(3), 853-863 (1985).

[4]Işcan, M.Y., Loth, S.R. & Wright, R.K. Age estimation from the rib by phase analysis: white males. *Journal of Forensic Science*, 29(4), 1094-1104 (1984).

[5]Verzeletti, A., Cassina, M., Micheli, L., Conti, A., & De Ferrari, F. Age estimation from the rib by components method analysis in white males. *The American Journal of Forensic Medicine and Pathology*, *31*(1), 27-33 (2010).

[6]Muñoz, A., Maestro, N., Benito, M., Sánchez, J. A., Márquez-Grant, N., Trejo, D., & Ríos, L. Sex and age at death estimation from the sternal end of the fourth rib. Does Íşcan's method really work? *Legal Medicine*, *31*, 24-29 (2018).
[7]Meindl, R.S. & Lovejoy, C.O. Ectocranial suture closure: A revised method for the determination of skeletal age at death based on the lateral-anterior sutures. *American Journal of Physical Anthropology*, 68(1), 57-66 (1985).
[8]Todd, T.W. Age changes in the pubic bone. I. The male white pubis. *American Journal of Physical Anthropology,* 3(3), 285-334 (1920).
[9]Brooks, S. & Suchey, J.M. Skeletal age determination based on the os pubis: a comparison of the Acsádi-Nemeskéri and Suchey-Brooks methods. *Human Evolution*, 5(3), 227-238 (1990).
[10]Lovejoy, C.O., Meindl, R.S., Pryzbeck, T.R. & Mensforth, R.P. Chronological metamorphosis of the auricular surface of the ilium: a new method for the determination of adult skeletal age at death. *American Journal of Physical Anthropology*, 68(1), 15-28 (1985).
[11]Trotter, M. & Gleser, G.C. Estimation of stature from long bones of American Whites and Negroes. *American Journal of Physical Anthropology*, 10(4), 463-514 (1952).
[12]Bass, W.M. *Human Osteology: A Laboratory and Field Manual.* (Columbia: Missouri Archaeological Society, 2006).
[13]Robins, G. & Shute, C.C.D. Predynastic Egyptian stature and physical proportions. *Human Evolution*, 1(4), 313-324 (1986).
[14]Raxter, M.H., Ruff, C.B., Azab, A., Erfan, M., Soliman, M. & El-Sawaf, A. Stature estimation in ancient Egyptians: a new technique based on anatomical reconstruction of stature. *American Journal of Physical Anthropology*, 136(2), 147-155 (2008).
[15]White, T.D., Black, M.T. & Folkens, P.A. *Human Osteology, Third Edition*. (New York: Academic Press, 2012).
[16]Flouri, D.E., Alifragki, A., García-Donas, J.G. & Kranioti, E.F. Ancestry estimation: advances and limitations in forensic applications. *Research and Reports in Forensic Medical Science*, 12, 13-24 (2002).
[17]Turner, C.G., Nichol, C.R., & Scott, G.R. Scoring procedures for key morphological traits of the permanent dentition: the Arizona State University dental anthropology

system. In: Kelley, M.A., Larsen, C.S. (Eds.), *Advances in Dental Anthropology*. Wiley-Liss, New York, 13- 32, 1991).

[18]Scott, G.R. & Irish, J.D. *Human Tooth Crown and Root Morphology. The Arizona State University Dental Anthropology System*. (Cambridge University Press, Cambridge, 2017).

[19]Howells, W. W. *Skull Shapes and the Map: Craniometric Analyses in the Dispersion of Modern* Homo. Peabody Museum of Archaeology and Ethnology, Vol. 79. (Cambridge: Harvard University Press, 1989).

[20]Irish, J.D., Morez, A., Girdland Flink, L., Phillips, E.L. & Scott, G.R. Do dental nonmetric traits actually work as proxies for neutral genomic data? Some answers from continental-and global-level analyses. *American Journal of Physical Anthropology,* 172(3), 347-375 (2020).

[21]Scott, G.R., Pilloud, M.A., Navega, D., d'Oliveira, J., Cunha, E. & Irish, J.D. rASUDAS: A new web-based application for estimating ancestry from tooth morphology. *Forensic Anthropology*, 1(1), 18-31 (2018).

[22]Wright, R. Guide to Using the CRANID programs CR6bIND: For Linear and Nearest Neighbours Discriminant Analysis (2012). Accessed online 1 November 2022 https://www.scribd.com/document/324417767/CRANID6b-ManuaL-1-pdf.

[23]Ortner, D.J. & Putschar, W. I*dentification of Paleopathological Conditions in Human Skeletal Remains*. (Washington: Smithsonian Institution Press, 1985).

[24]Aufderheide, A.C. & Rodríguez-Martín, C. *The Cambridge Encyclopedia of Human Paleopathology.* (Cambridge: Cambridge University Press, 1998).

[25]Capasso, L., Kennedy, K.A.R. & Wilczak, C.A. *Atlas of Occupational Markers on Human Remains*. (Teramo, Italy: Edigrafital S.p.A., 1998).

[26]Burnett, S.E. Crown wear: identification and categorization. In: J.D. Irish & G.R. Scott (Eds.) *A Companion to Dental Anthropology*. (New York: Wiley-Blackwell. 415-432, 2016).

[27]Kennedy, K.A.R. Skeletal markers of occupational stress. In: M.Y. Iscan & K.A.R. Kennedy (Eds.) *Reconstruction of Life from the Skeleton*. (New York: Alan R. Liss. 129-160, 1989).

[28]Hawkey, D.E. & Merbs, C.F. Activity-induced musculoskeletal stress markers (MSM) and subsistence strategy changes among ancient Hudson Bay Eskimos. *International Journal of Osteoarchaeology*, 5, 324-338 (1995).

[29]Abarca-Labra, V., Herrera-Soto, M. J., Flores-Alvarado, S., Ulloa-Velásquez, C., Urrutia- Álvarez, C., Falabella-Gellona, F., & Sanhueza-Riquelme, L. Exploring physical activity in Central Chile during the Early Ceramic Period and Late Intermediate Period (200–1450 CE). *American Journal of Biological Anthropology*, *177*(4), 658-668 (2022).

[30]Carballo-Pérez, J., Sánchez-Cañadillas, E., Arnay-de-la-Rosa, M., Hernández-Marrero, J.C., & González-Reimers, E. Quotidian lives on isolated bodies: Entheseal changes and cross- sectional geometry among the aboriginal population of La Gomera (ca. 200–1500 AD, Canary Islands). *International Journal of Osteoarchaeology*, 31(3), 366-381 (2012).

[31]Bucchi, A., Luengo, J., Del Bove, A., & Lorenzo, C. Insertion sites in manual proximal phalanges of African apes and modern humans, *American Journal of Physical Anthropology*, 173(3), 556-567 (2020).

[32]Refai, O. Entheseal changes in ancient Egyptians from the pyramid builders of Giza—Old Kingdom, *International Journal of Osteoarchaeology,* 29(4), 513-524 (2019).

[33]Alves-Cardoso, F. & Assis, S. Exploring “wear and tear” of joints and “muscle function” assumptions in skeletons with known occupation at death. *American Journal of Physical Anthropology,* 175(3), 689-700 (2021).

[34]Wallace, I.J., Riew, G.J., Landau, R., Bendele, A.M., Holowka, N.B., Hedrick, T.L., Konow, N., Brooks, D.J. & Lieberman, D.E. Experimental evidence that physical activity inhibits osteoarthritis: Implications for inferring activity patterns from osteoarthritis in archeological human skeletons. *American Journal of Biological Anthropology,* 177(2), 223-231 (2022).

[35]Shahar, D. & Sayers, M.G. Prominent exostosis projecting from the occipital squama more substantial and prevalent in young adult than older age groups. *Scientific Reports,* 8(1), 1-7 (2018).

[36]Grubmuller, A., Botkin, C., Frye, S., Muzaffr, R. & Osman, M. Occipital exostosis resulting from excessive screen time. J*ournal of Nuclear Medicine*, 61(Suppl. 1), 3047 (2020).

[37]Hawkey, D.E. & Street, S.R. Activity-induced stress markers in prehistoric human remains from the eastern Aleutian Islands. *American Journal of Physical Anthropology*, Suppl. 14, 89 (1992).

[38]Épron, L. & Daumas, F. *Le Tombeau de Ti Fasc.1, les Approaches de la Chapelle*. (Cairo: Impr. De l'Institut francais d'archéologie orientale, 1939).

[39]Doherty, S.K. *The Origins and Use of the Potter's Wheel in Ancient Egypt*. (PhD Thesis, Cardiff University, United Kingdom, 2013).

[40]Newberry, P.E. *Beni Hasan Part 1*. (London: Kegan Paul, Trench, Trubner & Co., Ltd., 1893).

[41]Davies, N.de.G. *The Tomb of Ken-Amun at Thebes, Vols. I-II*. (New York: The Metropolitan Museum of Art, 1930).

[42]Garstang, J. *The Burial Customs of Ancient Egypt*. (Liverpool: University of Liverpool, Institute of Archaeology, 1907).

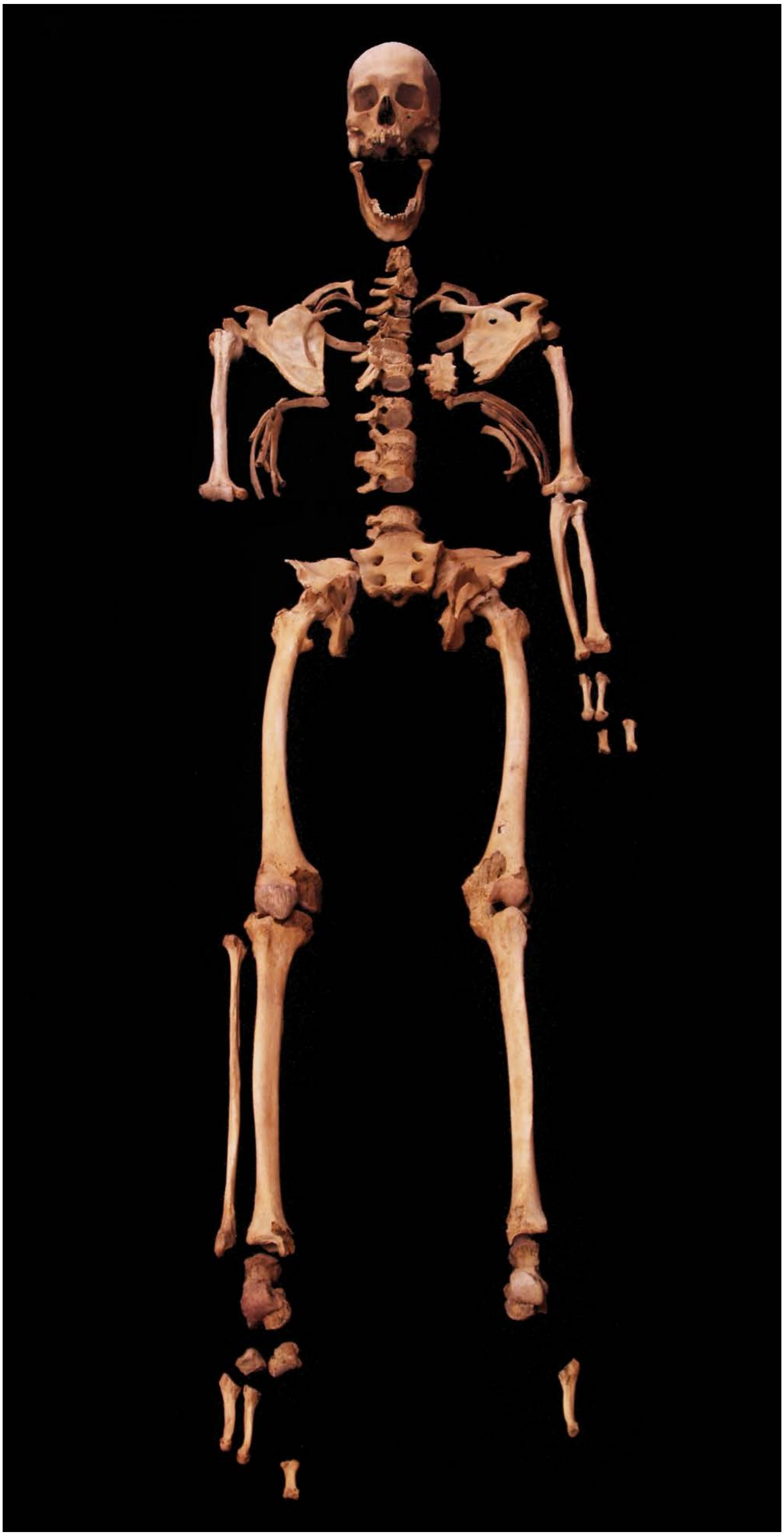

Figure S2.1. The Nuwayrat 50•33 skeleton (photo J.D. Irish).

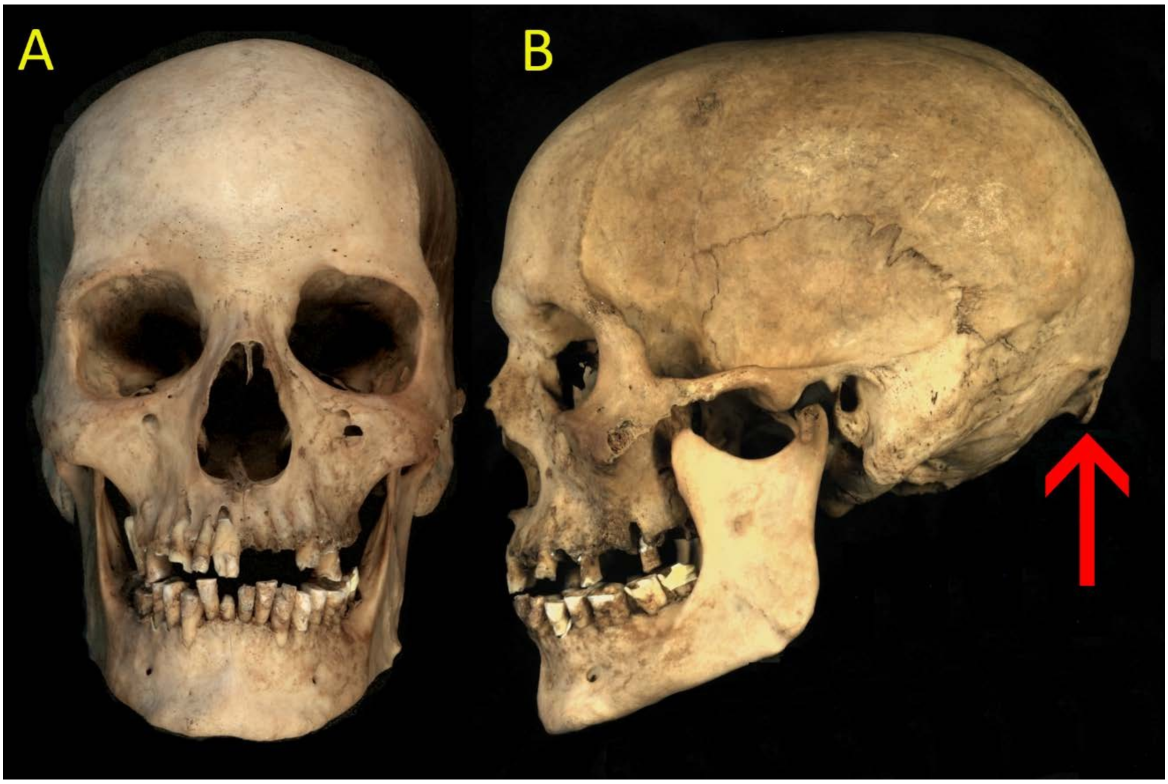

Figure S2.2. A) Front view of the Nuwayrat skull. B) Left side of the skull. Arrow indicates enlarged external occipital protuberance. See text for details (photo J.D. Irish).

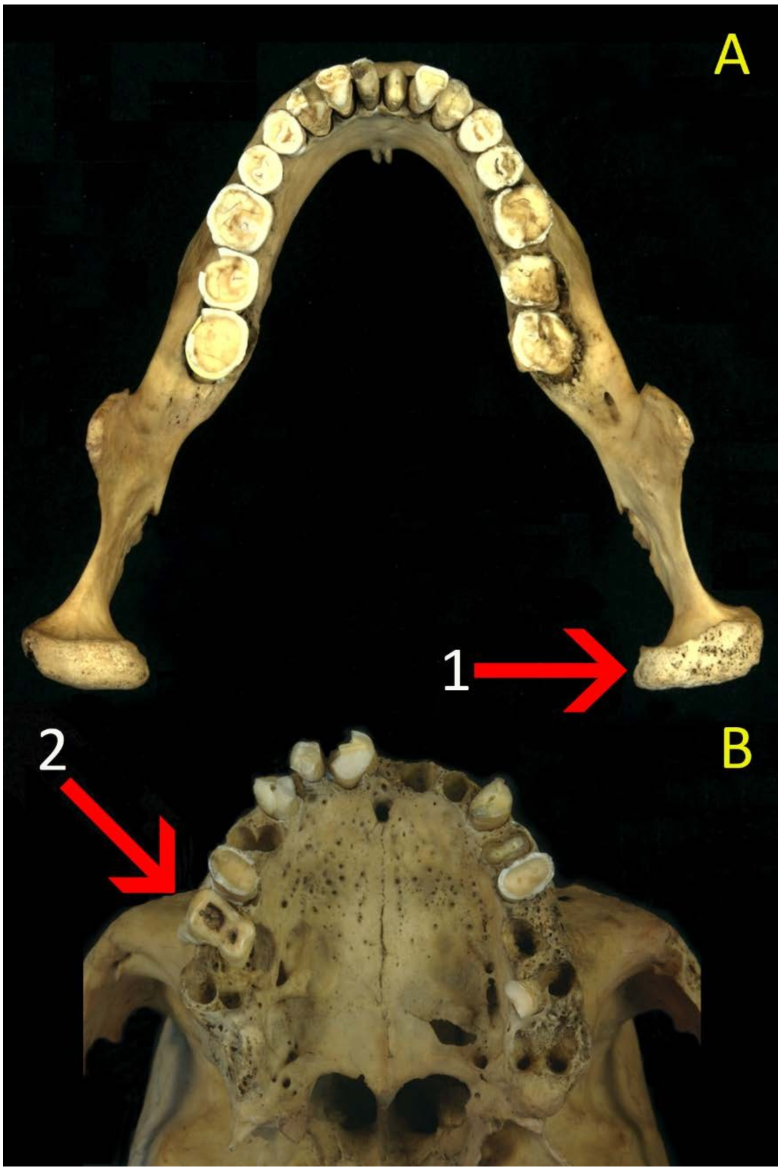

Figure S2.3. A) Mandible and dentition. Note crown wear, most noticeably on the right molars. Arrow 1 indicates advanced osteoarthritis of the temporomandibular joint (DJD). B) Maxilla and dentition. Arrow 2 points to the extremely worn upper right first molar, with accompanying large alveolar abscess. Note damaged and missing teeth. See text for details (photo J.D. Irish).

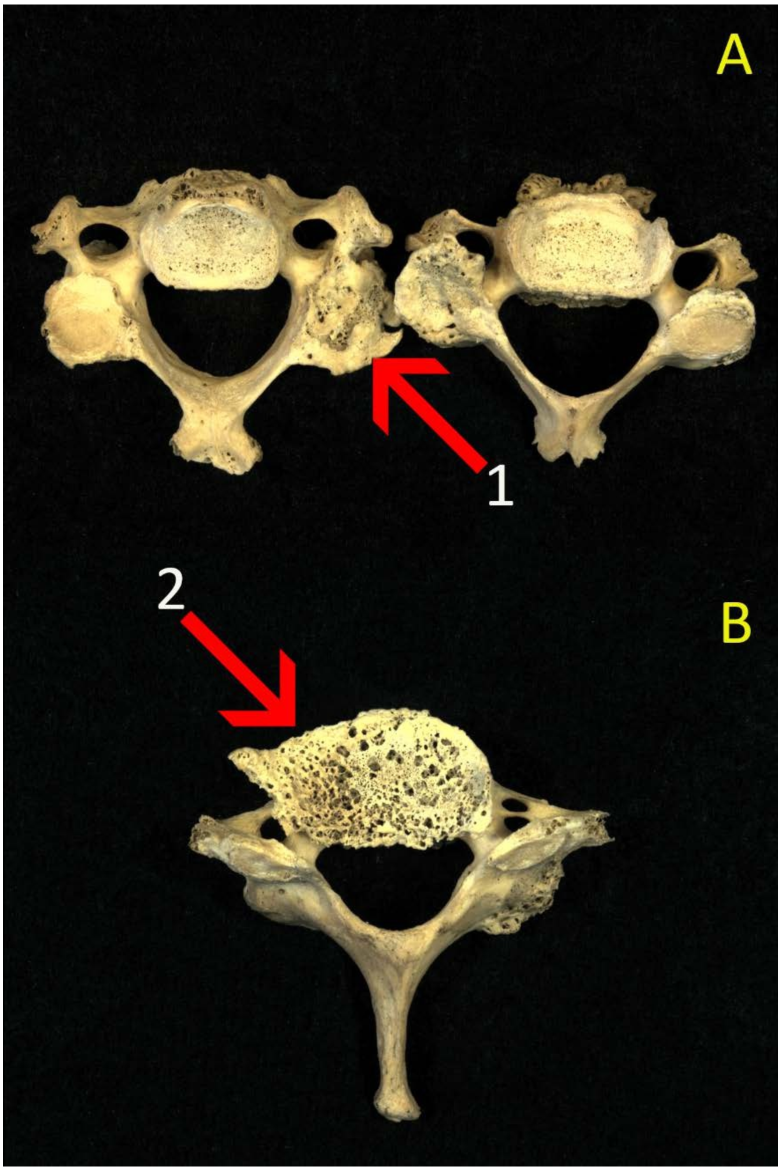

Figure S2.4. A) Third (inferior view) and fourth (superior view) cervical vertebrae. Arrow 1 points toward the osteoarthritis of the articular facets (DJD). Some osteophyte development is also evident on the bodies (DDD). B) Sixth cervical vertebra (superior view). Arrow 2 indicates the extreme osteoarthritis of the C7 body (DDD). The right articular facet is affected as well. See text for details (photo J.D. Irish).

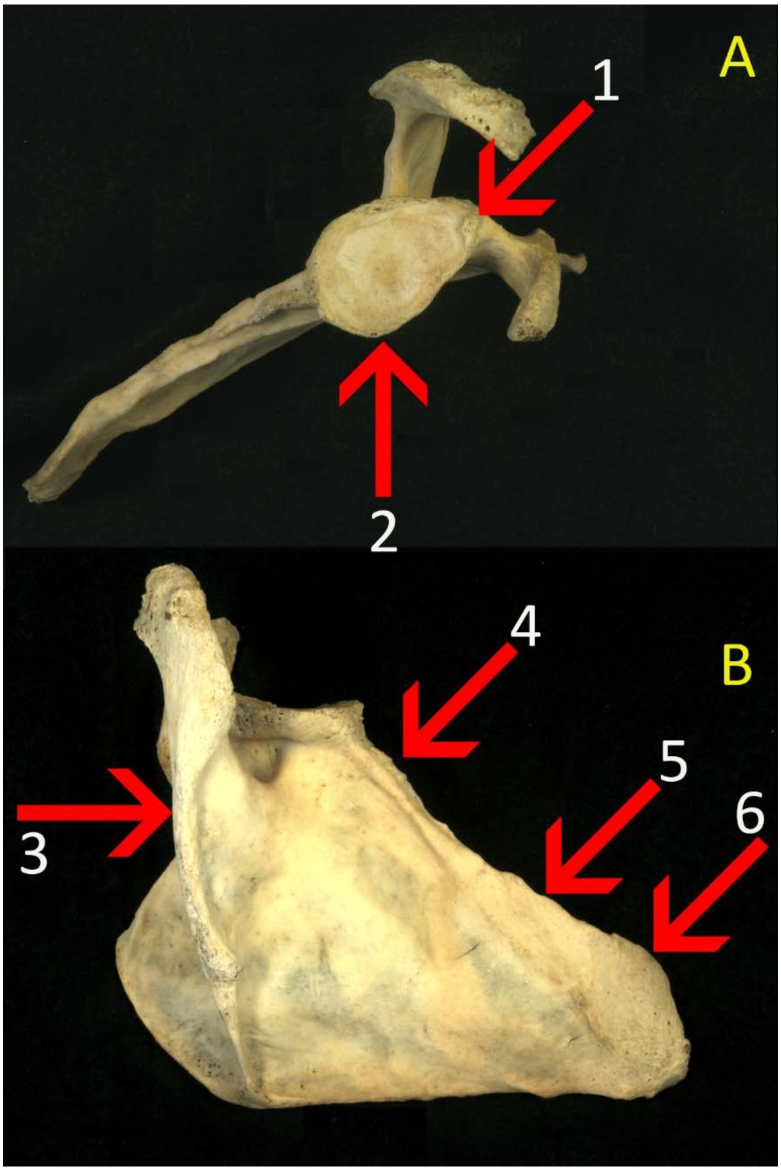

Figure S2.5. A) Lateral view of the right scapula. Arrow 1 points to a supraglenoid articular facet, and 2 toward marginal osteophytosis around the glenoid fossa. B) Dorsal view of the right scapula. The remaining arrows indicate well developed muscle origins as follows: 3, deltoid muscle on the spine and lateral acromion process; 4, long head of the triceps brachii; 5, teres minor; and 6; teres major. See text for details (photo J.D. Irish).

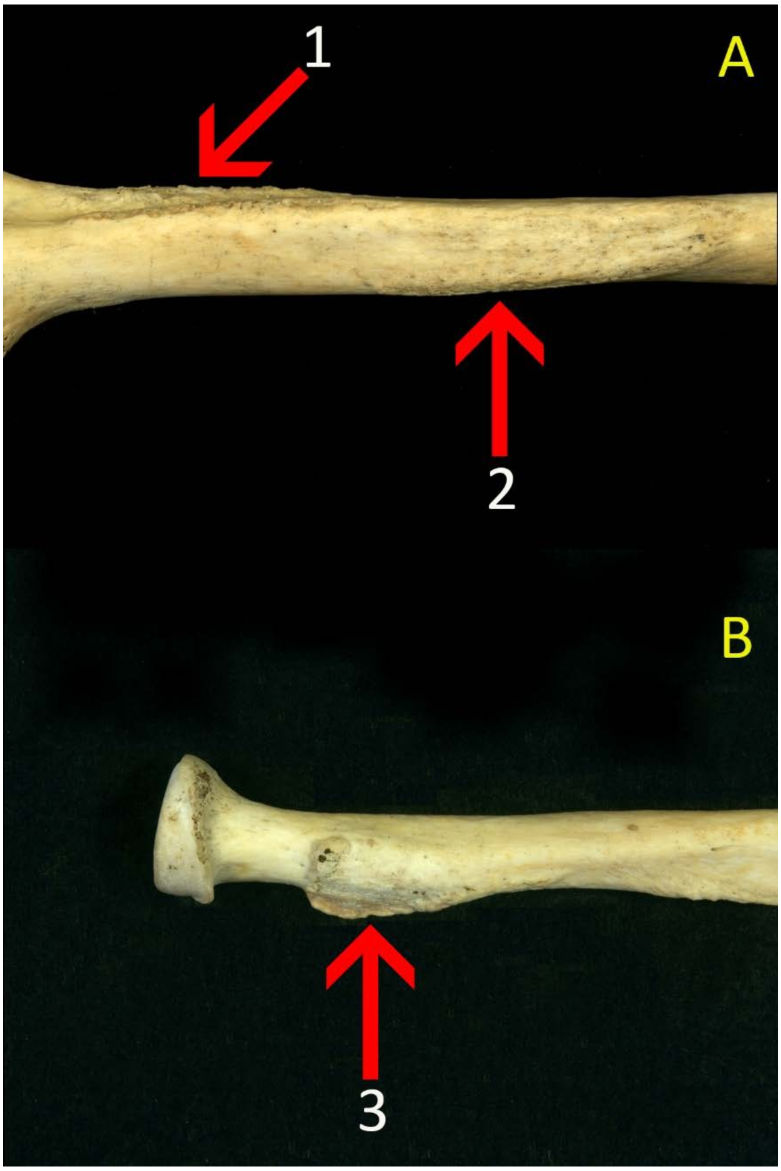

Figure S2.6. A) Posterior view of the right humerus. Arrow 1 indicates raised insertions for the teres major, pectoralis major, and latissimus dorsi muscles, and 2 the deltoid tuberosity. B) Anterior view of left radius, with Arrow 3 indicating insertion for biceps brachii muscle on the radial tuberosity. See text for details (photo J.D. Irish).

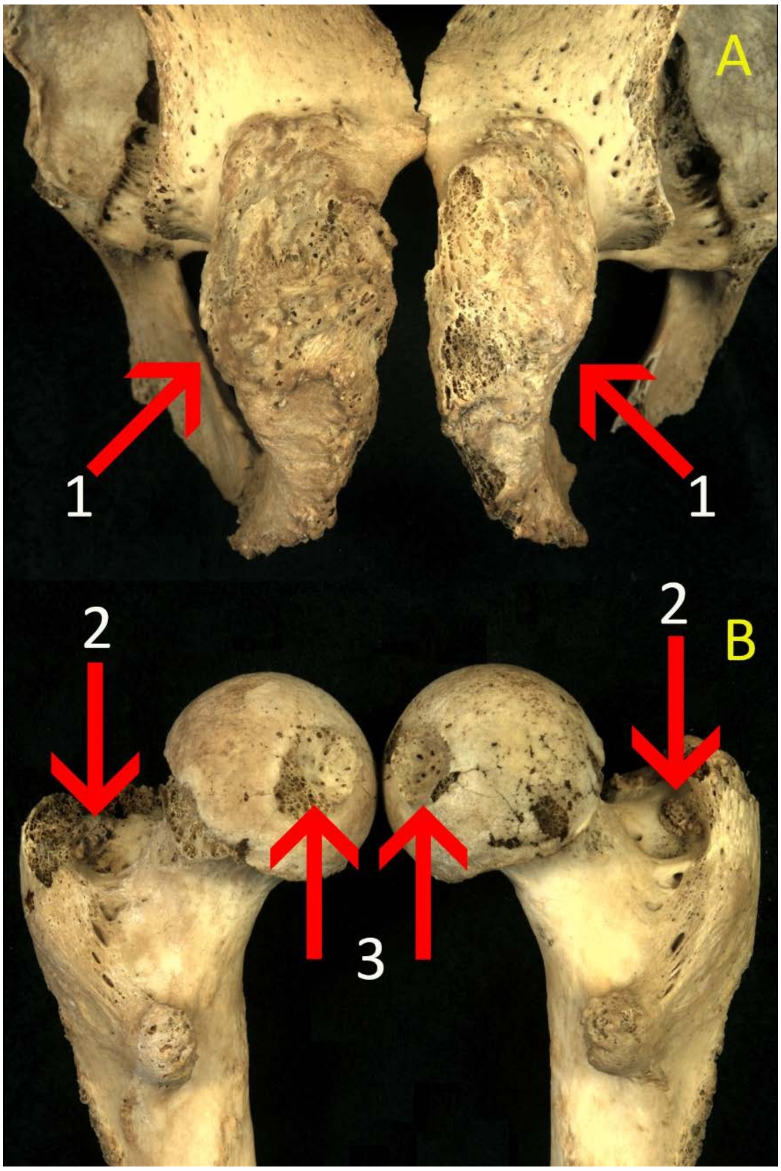

Figure S2.7. A) Dorsal view of left and right innominates of the pelvis, with Arrow(s) 1 indicating osteitis of the ischia, with left side appearing to be more affected. B) Proximal femora evidencing exostoses in the trochanteric fossae, which are insertion points for the obturator externus muscle [Arrow(s) 2] and bilateral expansion of the fovea capitis, to which attaches the ligamentum teres [Arrow(s) 3]. See text for details (photo J.D. Irish).

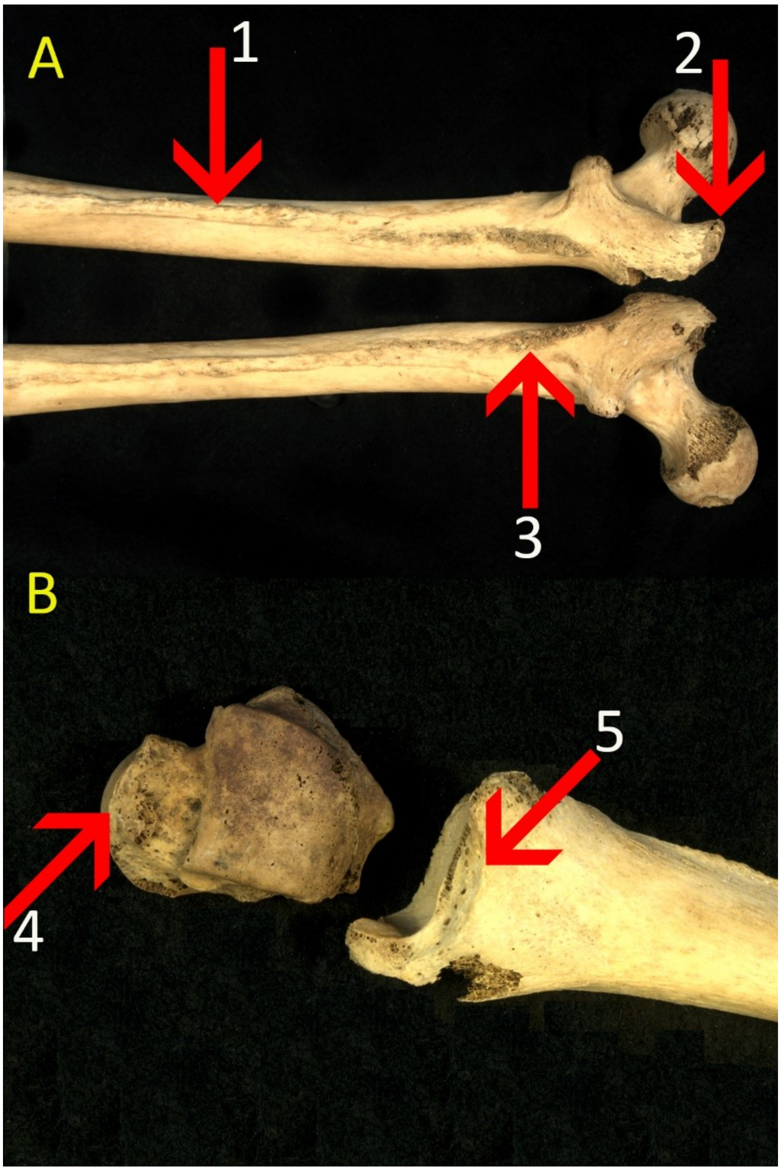

Figure S2.8. A) Posterior view of right and left femora. Arrows indicate substantial development of the: 1, linea apsera for the adductor longus; 2, greater trochanter for insertion of the gluteus medius and minimus muscles; and 3, gluteal tuberosity for gluteus maximus attachment. B) Right talus and distal tibia. Arrows 4 and 5 point to corresponding facets on the two bones from hyper- dorsiflexion. See text for details (photo J.D. Irish).

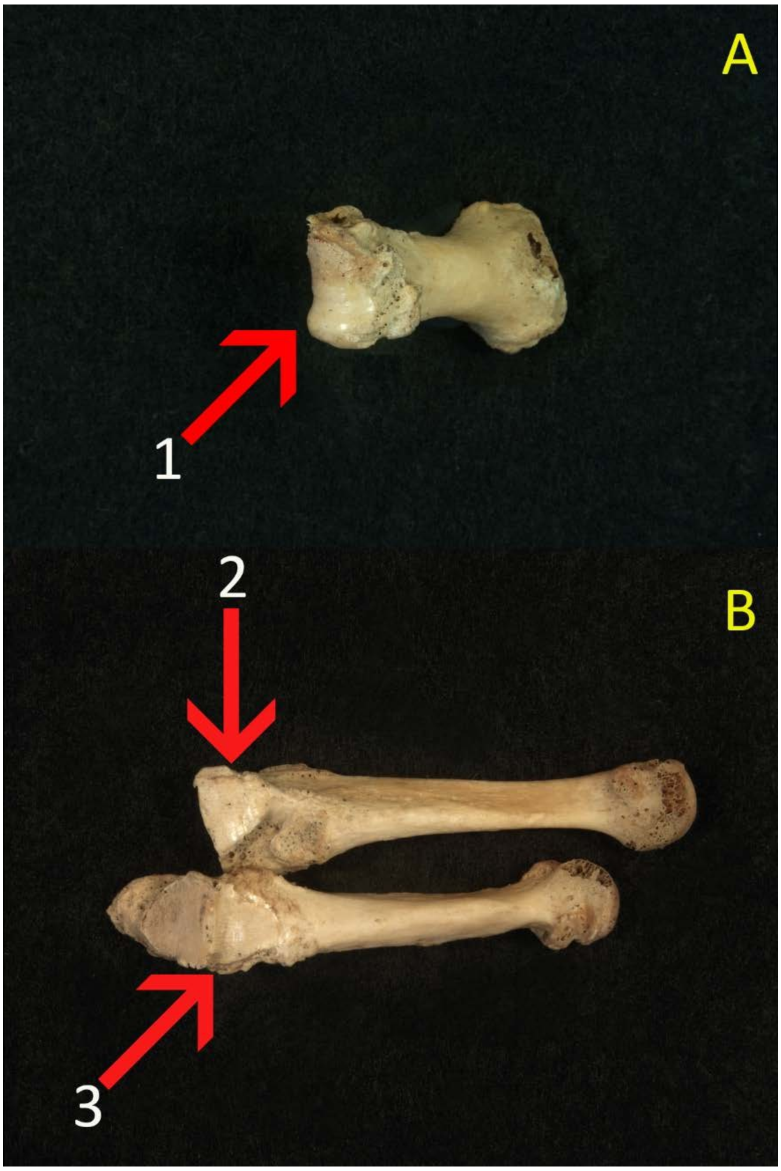

Figure S2.9. A) Distal end of the proximal phalange of right hallux. Arrow 1 indicates eburnation and osteophyte development (DJD). B) Right fourth and fifth metatarsals evidencing eburnation on the adjoining facets (Arrows 2-3). See text for details (photo J.D. Irish).

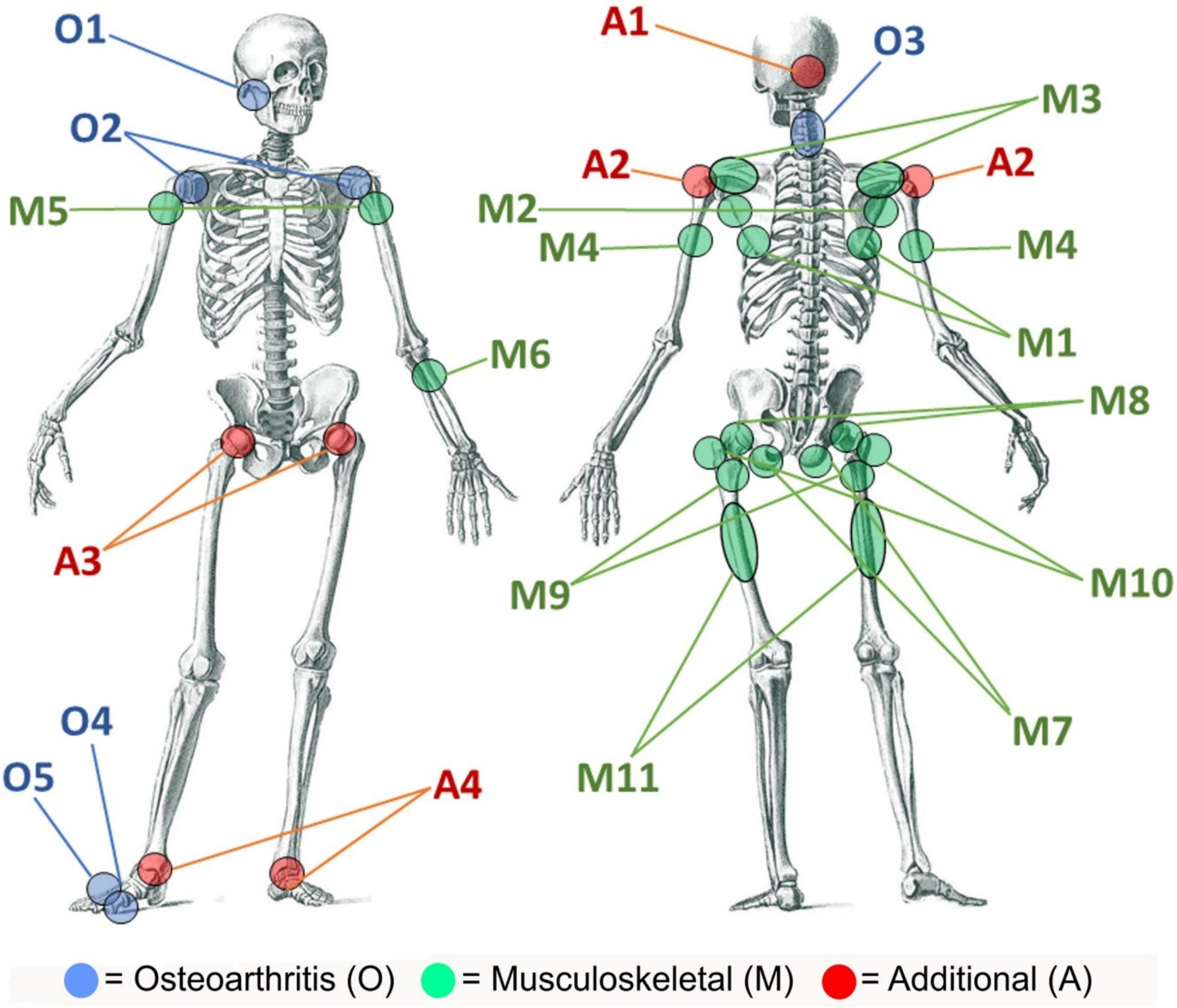

Figure S2.10. Location of the skeletal markers of physical stress presented and quantified in Tables S2.4-S2.6. The codes are described in the latter tables. See main text for details. Skeleton images are from https://pixabay.com/illustrations/human-skeleton-skeletons-anatomy-5500722/.

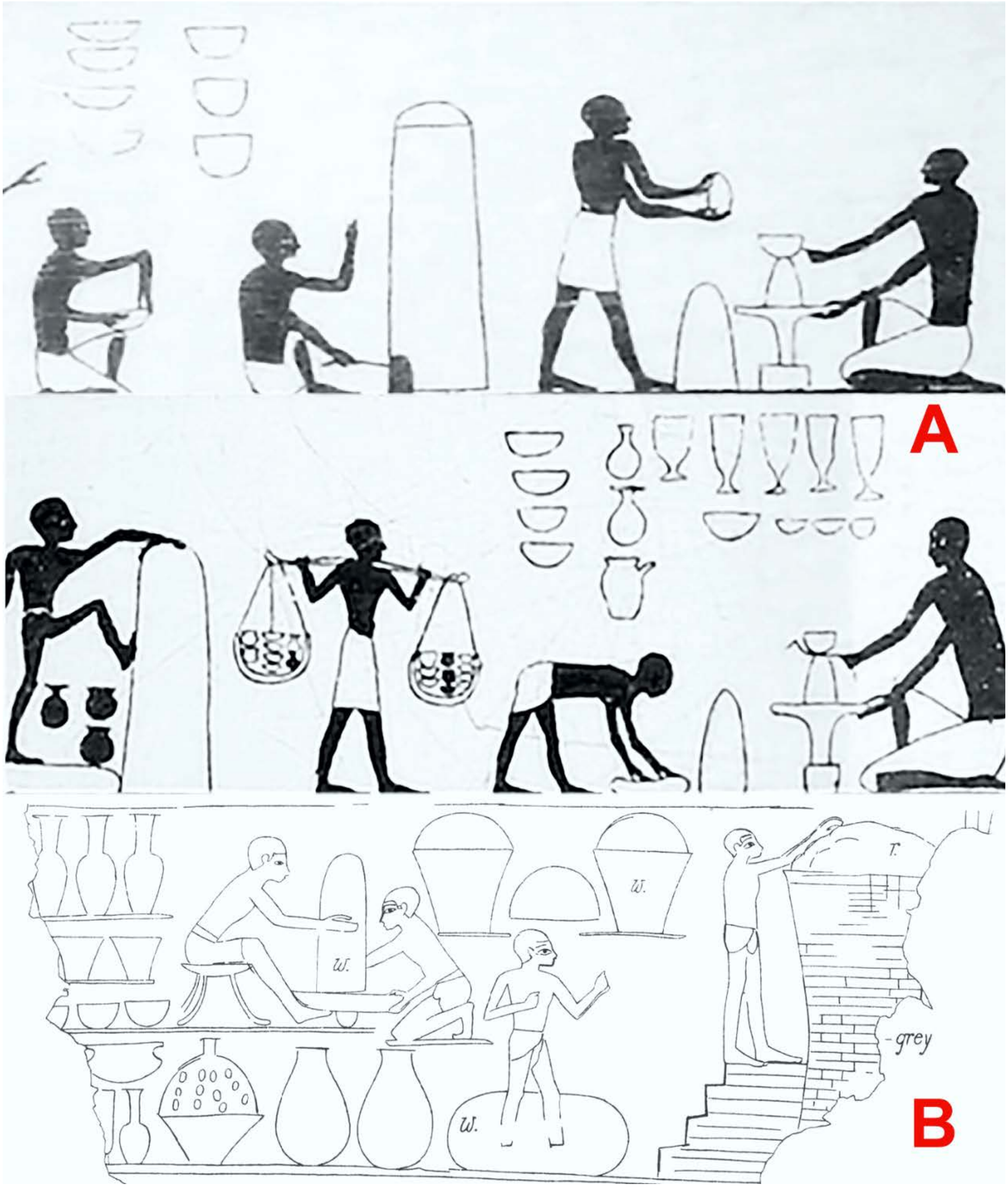

Figure S2.11. A) Drawings of two sections in a longer panel of a pottery workshop from a 12th Dynasty (c. 1938-1773 BCE) wall painting in the tomb of Amenemhat I at Beni Hasan, near Nuwayrat (in Newberry[40]), with positions required by potters for the various tasks illustrated. B) Drawing of painting from a mid-18th Dynasty (1427-1401 BCE) tomb showing the same workshop process (in Davies[41]). See text for details.

Table S2.1. Stature estimates of the Nuwayrat individual based on long bone dimensions.

		White[a]	Black[b]	Ancient Egyptian[c]		
**Element**	**Length cm**	**Stature cm**	**Stature cm**	**Formula**	**SEE[d]**	**Stature cm**
Humerus	28.90	159.00	156.00	2.594 (hum) + 83.85	4.218	158.82
Radius	22.60	164.00	159.00	2.641 (rad) + 100.91	3.731	160.60
Ulna	24.90	166.00	160.00			
Femur	41.40	160.00	158.00	2.257 (fem) + 63.93	3.218	157.37
Tibia[e]	35.40	168.00	163.00	2.552 (tib) + 70.18	3.060	160.52
Fibula	34.10	163.00	160.00			
Femur & Tibia[e]	76.80	164.00	159.00	1.276 (fem + tib) + 60.64	2.900	158.64
Humerus & Radius	51.50			1.456 (hum + rad) + 83.76	3.353	158.74
Mean Stature		163.43	159.29			159.12
Correction for Age[f]		-0.06	-0.06			-0.06
**Final Estimate**		**163.37**	**159.23**			**159.06**

[a]Based on regression formulae of American Whites from Trotter and Gleser[11], with tables from Bass[12].

[b]Based on regression formulae of American Blacks from Trotter and Gleser[11], with tables from Bass[12].

[c]Based on reconstruction of ancient Egyptian skeletal remains and American Whites and Blacks, with new regression formulae from Raxter et al.[14].

[d]Standard errors of estimates (SEE) from Raxter et al.[14].

[e]All long bone measurements are maximum length, with exception of the tibia measured to the lateral condyle.

[f]Suggested stature estimation correction in adult individuals >30 years of age (Bass[12]; Raxter et al.[14]).

Table S2.2. The 15 of 32 traits that could be scored in the Nuwayrat dentition for rASUDAS program.

Trait[a]	Score	Trait	Score
Winging (UI1)	0	Premolar multiple lingual cusps (LP2)	2
Shoveling (UI1)	0-1	Enamel extensions (LM1)	2-3
Shoveling (UI2)	NA[b]	Cusp 6 (LM1)	NA
Double shoveling (UI1)	NA	Cusp 6 (LM2)	NA
Tuberculum dentale (UI1)	NA	Cusp 7 (LM1)	0
Interruption grooves (UI2)[c]	0	Deflecting wrinkle (LM1)	NA
Bushman Canine (UC)	NA	Protostylid (LM1)	NA
Tuberculum dentale (UC)	NA	Groove pattern (LM2)	X or +
Carabelli's trait (or cusp) (UM1)	NA	4-cusped molar (LM1)	NA
Cusp 5 (UM1)	NA	4-cusped molar (LM2)	NA
Cusp 5 (UM2)	NA	Upper premolar root no (UP1)	1
Enamel extensions (UM1)	NA	Upper second molar root no (UM2)	1 - 2
Hypocone (UM2)	NA	Lower canine root no (LC)	1
PRM (UM3)	0	Tomes' root (LP1)	0- 3
Distal accessory ridge (LC)	NA	3-rooted lower first molar (LM1)	1 - 2
Premolar multiple lingual cusps (LP1)	2- 3	1-rooted lower second molar (LM2)	1

[a]Traits and scoring defined in Turner et al.[17], Scott and Irish[18], and Scott et al.[21].
[b]NA=trait not scorable.
[c]UI2 not present, so UI1 score substituted.

Table S2.3. The 29 craniometric measurements in mm of the Nuwayrat cranium used in CRANID[a] program.

Code	Measurements[b]	mm
GOL	Glabello-occipital length	190
NOL	Nasio-occipital length	187
BNL	Basion-nasion length	101
BBH	Basion-bregma height	130
XCB	Maximum cranial breadth	130
XFB	Maximum frontal breadth	113
AUB	Biauricular breadth	111
ASB	Biasterionic breadth	105
BPL	Basion-prosthion length	95
NPH	Nasion-prosthion height	63
NLH	Nasal height	50
OBH	Orbit height	31
OBB	Orbit breadth	37
JUB	Bijugal breadth	109
NLB	Nasal breadth	25
MAB	Palate breadth, external	64
ZMB	Bimaxillary breadth	93
SSS	Zygomaxillary subtense	26
FMB	Bifrontal breadth	94
NAS	Nasio-frontal subtense	19
EKB	Biorbital breadth	92
DKB	Interorbital breadth	24
WMH	Cheek height	20
FRC	Nasion-bregma chord	110
FRS	Nasion-bregma subtense	27
PAC	Bregma-lambda chord	122
PAS	Bregma-lambda subtense	28
OCC	Lambda-opisthion chord	92
OCS	Lambda-opisthion subtense	24

[a]CRANID program from Wright[22].

[b]Codes and measurements from Howells[19].

Table S2.4. Scores for osteoarthritis markers of physical stress mentioned in the text[a]

Code[b]	Location(s)	Osteophytosis (Lipping)[c]						Porosity[d]						Eburnation[e]			
		**Left**				**Right**		**Left**				**Right**		**Left**		**Right**	
O1	Temporomandibular joint	0				3		1				3		0		1	
O2	Glenoid fossa	2				2		1				1		0		0	
O3	Cervical vertebrae	Facet		Body		Facet		Facet		Body		Facet		Facet		Facet	
		Sup	Inf	Sup	Inf	Sup	Inf	Sup	Inf	Sup	Inf	Sup	Inf	Sup	Inf	Sup	Inf
	C3	3	3	1	1	1	2	3	3	1	1	1	1	2	0	0	2
	C4	3	2	1	3	2	3	3	2	1	3	1	3	0	2	2	1
	C5			3	4					3							
	C6			4	3						3						
	C7	1	1	3	3	1	3	0	1	3	3	0	3	0	0	0	0
O4	Metatarsals 4-5					2						1				2	
O5	Hallux proximal phalange					3						3				3	

[a]Based on standardized scoring system in Buikstra and Ubelaker[1]
[b]Codes pertain to locations of these OA markers (see text for details) throughout the skeleton in Figure S2.10.
[c]Osteophytosis grades 0=none, 1=barely discernible, 2=sharp ridge, sometimes with spicules (bony spurs), 3=extensive spicule formation, and 4=ankylosis (fusion).
[d]Porosity, 0=none, 1=pinpoint, 2=coalesced, and 3=pinpoint and coalesced.
[e]Eburnation, 0=none, 1=barely discernible, 2=polish, and 3=polish with groove(s) (Buikstra and Ubelaker[1]).

Table S2.5. Scores for musculoskeletal markers of physical stress mentioned in the text[a]

Code[b]	Location(s)	Robusticity (Origin)[c]		Robusticity (Insertion)[d]	
		Left	Right	Left	Right
M1	Scapula thickening of margin for teres major and teres minor	2	2		
M2	Scapula superior lateral margin for long head of triceps brachii	3	3		
M3	Scapula dorsal spine and lateral acromion process for deltoid	3	3		
M4	Humerus proximal posterior deltoid tuberosity			3	3
M5	Humerus proximal anterior for teres major, pectoralis major, and latissimus dorsi			3	3
M6	Radius proximal anterior radial tuberosity for bicips brachii			3	
M7	Ischium for hamstrings[e]	3	3		
M8	Femur proximal superior throchanteric fossa for obturator externus			3	3
M9	Femur proximal posterior gluteal tuberosities for gluteus maximus			3	3
M10	Femur proximal posterior greater trochanter for for gluteus medius and minimus			3	3
M11	Femur posterior linea aspera for adductor longus			3	3

[a]Based on standardized scoring system in Hawkey and Merbs[2].
[b]Codes pertain to locations of these MSMs (see text for details) throughout the skeleton in Figure S2.10.
[c]Robusticity grading for muscle origin sites 1=slight rounding of the cortex, 2=uneven cortical surface with mound shaped elevation, and 3=distinct sharp crests/ridges.
[d]Robusticity grading for muscle insertion sites 1=slight indentation with no surrounding bony margin, 2=roughened with defined margin, and 3=deep indentation with clearly defined margin (Hawkey and Merbs[2]).
[e]Hamstring group composed of multiple muscles originating on each ischium (See text).
[b]Codes pertain to locations of these MSMs (see text for details) throughout the skeleton in Figure S2.10.
[c]Robusticity grading for muscle origin sites 1=slight rounding of the cortex, 2=uneven cortical surface with mound shaped elevation, and 3=distinct sharp crests/ridges.
[d]Robusticity grading for muscle insertion sites 1=slight indentation with no surrounding bony margin, 2=roughened with defined margin, and 3=deep indentation with clearly defined margin (Hawkey and Merbs[2]).
[e]Hamstring group composed of multiple muscles originating on each ischium (See text).

Table S2.6. Scores for additional markers of physical stress mentioned in the main text

Code[a]	Location(s)	Robusticity[b]		
		Left	Medial	Right
A1	Cranium posterior, external occipital protuberance for nuchal ligament		3	
A2	Scapula glenoid fossa, supraglenoid articular facet	NA		NA
A3	Femur, head for ligamentum teres		3	
A4	Tibia distal anterior & talus superior head	NA		NA

[a]Codes pertain to locations of these additional markers (see text for details) throughout the skeleton in Figure S2.10.

[b]Adapted from scoring system in Hawkey and Merbs[28] (see Table S2.5) to permit some level of quantification for ligament attachment expression.

SI 3. Facial reconstruction of the Nuwayrat individual

*Corresponding author: C.M.Wilkinson@ljmu.ac.uk (C. W.)

Context

The cultural and social interpretation of archaeological remains frequently involves the facial depiction of people from the past[1], as this helps the public connect with their ancestors, perpetuating affective bonds and sustained interest[2]. In this way, facial depictions have contributed to changing our understanding of historical figures[3–5] and ancient migration or geographical paterns[6–8]. In addition, facial depiction thwarts the classification of human remains as museum objects/artefacts[9], whilst manifesting evidence and playing an active part in academic debate. However, although a facial depiction may be based upon established scientific knowledge, subjective interpretation will inevitably be utilised when there is insufficient evidence on facial appearance, and this may perpetuate confirmation bias[10].

Whilst research has shown good accuracy in relation to the prediction of face shape from skeletal structure[11,12], the addition of reliable colour, texture and detail is notoriously difficult to achieve. Psychology studies suggest that different hairstyles[13] and facial hair[14] can have an alarmingly strong effect upon facial appearance and recognition levels. In archaeological investigations, the most probable hairstyle, hair colour, skin colour and eye colour will typically be depicted based on contextual information. It is therefore important not to consider a facial depiction as a portrait or definitive image, as it can only visualise the available information and additional evidence could lead to a future iteration.

In this case, although DNA analysis suggested the most likely population of origin, there was no evidence in relation to eye colour, skin colour and hair colour. Therefore, the facial depiction was produced in black and white without hair or facial hair.

The craniofacial structure is directly related to function (mastication, respiration and protection of the brain) and the relationship between the bone and soft tissues is reciprocal and responsive[15,16]. Therefore, the principles of facial reconstruction are based on the theory that the shape of the soft tissues of the head are directly related to the skeletal morphology of the skull.

Craniofacial standards are based on early human dissection research supplemented by more recent clinical imaging, anthropometry and surface scan research. In this way, Whitnall[17] described in detail the relationship between the morphology of the eye and the orbital bones, specifically eyeball position, the palpebral ligaments of the eyelids, canthal angle/position and the lateral orbital (or Whitnall's) tubercle, and numerous research studies confirmed and augmented these standards[16,18–24].

Many studies have assessed the relationship between the configuration of the nasal soft tissues with the shape of the nasal aperture[18,25–37]. Early anatomical standards were confirmed in a clinical imaging study of living subjects[38], leading to the additional development of a nasal estimation method utilising three cranial measurements to predict six soft nose measurements. This method demonstrated a high level of accuracy in a subsequent blind test utilising a skeletal collection with related ante-mortem images[39]. Due to these standards, the nose is likely to be the most accurately predicted facial feature in facial reconstruction.

Orthodontic and anatomical literature demonstrates that mouth morphology is related to dental occlusion[40–46], dental pattern[21,47,48] and facial profile[18,49], and standards even exist to predict mouth shape for edentulous skulls[50]. Despite these standards, the exact shape of the vermillion line is difficult to achieve, and accuracy studies suggest that lip shape is one of the most error-prone areas of reconstruction[46].

There have been some studies [18,51,52] related to the prediction of ear morphology, but this feature remains the least predictable from skeletal interpretation.

Facial reconstruction utilises tissue depth data as guidance for the soft tissues of the face. Throughout the 20th century clinical imaging methods have been utilised to measure living subjects, including craniographs, Computed Tomography, Magnetic Resonance Imaging and ultrasound. More recent advances in the 21st century have included the use of low dose Cone-Beam Computed Tomography[53,54]. There are many sets of data available from differentin vivo ethnic groups across the world for use in craniofacial analysis. Collations of these datasets can be found in some publications[55,56] and websites[57].

Skull assessment

In this case, the cranium and mandible were intact with some missing teeth. The skull was

robust with strong muscle attachments visible at the occipital bone, temporal bone and mandible. The skull exhibited moderate brow ridges, gonial flaring, a square jawline and mental region, a strong supra-mastoid crest and large mastoid processes. These traits are consistent with a male skull.

The skull was dolichocephalic and exhibited a post-bregmatic depression.

The square orbits suggested slight upturned fissures (laterally), triangular eyebrows and normal eye ball protrusion.

The nasal spine and nasal bones suggested an upturned nose with rounded alae, a concave dorsal ridge and rounded tip.

The teeth demonstrated normal occlusion and suggested thicker lower than upper lip with a rounded lower lip shape. The canine fossae suggested nasolabial creases at the nasal portion.

The jaw line was square with visible gonial flaring. The mastoid processes suggested adherent ears.

The Nuwayrat skull data was collected using an Artec Space Spider 3D laser scanner and imported into Geomagic Freeform software. Egyptian male data[58] was used to estimate facial tissues at anatomical points across the skull surface. 24 mm eyeballs were set into the orbits at normal protrusion, taken as the cornea positioned 3.5 mm posterior to a tangent drawn from superior to inferior margins of the orbit[23].

The muscles of the head and neck were imported from a Face Lab database and remodelled to fit the skull following anatomical guidelines[1]. The details of the facial features were modelled with respect to the skull assessment. Adherent ears were modelled and attached to the sides of the head using the external auditory meatus to determine the position.

A skin layer was placed over the muscle structure to create the finished face. The layer mirrored the shape of the muscles and cranium below. During this process the tissue depth pegs were used as guides. The surface of the face was smoothed to achieve the final sculptural finish.

A frontal image of the facial reconstruction was imported into Adobe Photoshop and a facial

feature image database was utilised to add texture to the face. Facial features were chosen based on the parameters of the facial reconstruction and edited to match the appearance. In this way, a black and white facial depiction was produced (Fig. S3.1).

References

[1]Wilkinson, C. M. Facial reconstruction–anatomical art or artistic anatomy? *J. Anat.* **216**, 235–250 (2010).

[2]Buti, L., Gruppioni, G. & Benazzi, S. Facial reconstruction of famous historical figures: Between art and science. in *Studies in Forensic Biohistory: Anthropological Perspectives* (ed. Stojanowski. CM. & Duncan, W. N.) 191–212 (Cambridge University Press, Cambridge, 2016).

[3]Wilkinson, C. M. The Man Himself: The Face of Richard III. *The Ricardian Bulletin* (2013).

[4]Introna, F., Lauretti, C. & Wilkinson, C. M. San Nicola ed il suo volto. 29–50 (2018).

[5]Day, J. Facing the mummy: Physiognomy, facial reconstruction and the "delirious biographies" of Egyptian mummies. in *Proceedings of the 8th World Congress on Mummy Studies* (Rio de Janeiro, 2013).

[6]Gaspar, N., Valle, V. & Santos, R. V. The colour of the bones: Scientific narratives and cultural appropriations of "Luzia, " a prehistoric skull from Brazil. *Brazil. Mana* **5**, (2009).

[7]Shreeve, J. This face changes the human story. But how? *National Geographic* **228**, 30–57 (2015).

[8]Moon, P. The new face of Luzia and the Lagoa Santa people. *Agência Fapesp* htp://agencia.fapesp.br/the-new-face-of-luzia-and-the-lagoa-santa-people/29168 (2018).

[9]Charlier, P. Naming the body (or the bones): Human remains, anthropological/medical collections, religious beliefs, and restitution. *Clin. Anat.* **27**, 291–295 (2014).

[10]Wilkinson, C. M. Cognitive Bias and Facial Depiction from Skeletal Remains. *Bioarchaeology International* **4**, 1–14 (2021).

[11]Wilkinson, CM., Rynn, C., Peters, H., Taister, M., Kau, C. H., & Richmond, S. A blind accuracy assessment of computer-modeled forensic facial reconstruction using computed tomography data from live subjects. *Forensic Science, Medicine, and Pathology* **2**, 179–187 (2006).

[12]Lee, W.-J., Wilkinson, C. M. & Hwang, H.-S. An accuracy assessment of forensic computerized facial reconstruction employing cone-beam computed tomography from live subjects. *J. Forensic Sci.* **57**, 318–327 (2012).

[13]Wright, D. B. & Sladden, B. An own gender bias and the importance of hair in face recognition. *Acta Psychol.* **114**, 101–114 (2003).
[14]Lewis, M. B. Familiarity, target set and false positives in face recognition. *Eur. J. Cogn. Psychol.* **9**, 437–459 (1997).
[15]Kau, C. H. *et al.* Facial templates: a new perspective in three dimensions. *Orthod. Craniofac. Res.* **9**, 10–17 (2006).
[16]Rynn, C., Balueva, T. & Veselovskaya, E. Relationships between the skull and face. in *Craniofacial Identification* (eds. Wilkinson, C. M. & Rynn, C.) 193–202 (Cambridge University Press, 2012).
[17]Whitnall, S. E. *Anatomy of the Human Orbit and Accessory Organs of Vision: Krieger*. (Frowde and Hodder & Stoughton, 1921).
[18]Gerasimov, M. *The Reconstruction of the Face from the Basic Structure of the Skull.* (Nauka, Moscow, 1955).
[19]Gerasimov, M. M. *The Face Finder*. (Lippincot, New York, 1971).
[20]Couly, G., Hureau, J. & Tessier, P. Face: The anatomy of the external palpebral ligament in man. *Plast. Reconstr. Surg.* **60**, 473 (1977).
[21]Krogman, W. & Iscan, M. Y. *The Human Skeleton in Forensic Medicine*. (Charles C Thomas Publisher, Springfield, MO, 1986).
[22]Fedosyutkin, B. A., & Nainys, J. V. The relationship of skull morphology to facial features. in *Forensic analysis of the skull: craniofacial analysis, reconstruction, and identification.* (ed. Iscan, M. Y., & Helmer, R. P.) 199–213 (Wiley-Liss, New York, 1993).
[23]Wilkinson, C. M. & Mautner, S. A. Measurement of eyeball protrusion and its application in facial reconstruction. *J. Forensic Sci.* **48**, 12–16 (2003).
[24]Stephan, C. N. & Davidson, P. L. The placement of the human eyeball and canthi in craniofacial identification. *J. Forensic Sci.* **53**, 612–619 (2008).
[25]Tandler, J. U¨ber den Scha¨del Haydns. Miteilungen der. *Anthropologie Gesellschaft Wien* **39**, 260–280 (1909).
[26]Virchow, H. Die anthropologische Untersuchung der Nase. *Z Ethnol* **44**, 289–337 (1912).
[27]Selzter, A. P. The nasal septum: plastic repair of the deviated septum associated with a deflected tip. *Arch Otolaryngol* **40**, 433–444 (1944).
[28]Gray, L. The deviated septum – aetiology. 79, 567–575. *J Laryngol Otol* **79**, 567–575 (1965).
[29]Glanville, E. V. Nasal shape, prognathism and adaptation in man. *Am. J. Phys. Anthropol.* **30**, 29–37 (1969).

[30]Macho, G. A. An appraisal of plastic reconstruction of the external nose. *J. Forensic Sci.* **31**, 1391–1403 (1986).

[31]Mcclintock Robinson, J., Rinchose, D. J. & Zullo, T. G. Relationship of skeletal patern and nasal form. *Am J Orthod* **89**, 499–506 (1986).

[32]George, R. M. Anatomical and artistic guidelines for forensic facial reconstruction. in *Forensic analysis of the skull: craniofacial analysis, reconstruction, and identification* (ed. Iscan, M. Y., & Helmer, R. P.) 215–277 (Wiley-Liss, New York, 1993).

[33]Prokopec, M. & Ubelaker, D. H. Reconstructing the shape of the nose according to the skull. *Forensic Sci Commun* **4**, (2002).

[34]Schultz, A. H. Relation of the external nose to the bony nose and nasal cartilages in whites and negroes. *Am. J. Phys. Anthropol.* **1**, 329–338 (1918).

[35]Henneberg, M., Simpson, E. & Stephan, C. Human face in biological anthropology: Craniometry, evolution and forensic identification. in *The Human Face* 29–48 (Springer US, Boston, MA, 2003).

[36]Cerkes, N. The crooked nose: principles of treatment. *Aesthet. Surg. J.* **31**, 241–257 (2011).

[37]Davy-Jow, S. L., Decker, S. J. & Ford, J. M. A simple method of nose tip shape validation for facial approximation. *Forensic Sci. Int.* **214**, 208.e1–3 (2012).

[38]Rynn, C. Craniofacial approximation and reconstruction: tissue depth paterning and the prediction of the nose. (University of Dundee, Dundee, 2006).

[39]Rynn, C., Wilkinson, C. M. & Peters, H. L. Prediction of nasal morphology from the skull. *Forensic Sci. Med. Pathol.* **6**, 20–34 (2010).

[40]Rudee, D. A. Proportional profile changes concurrent with orthodontic therapy. *Am. J. Orthod.* **50**, 421–434 (1964).

[41]Roos, N. Soft-tissue profile changes in class II treatment. *Am. J. Orthod.* **72**, 165–175 (1977).

[42]Koch, R., Gonzales, A. & Wit, E. Profile and soft tissue changes during and after orthodontic treatment. *Eur. J. Orthod.* **1**, 193–199 (1979).

[43]Holdaway, R. A. A soft-tissue cephalometric analysis and its use in orthodontic treatment planning. Part I. *Am. J. Orthod.* **84**, 1–28 (1983).

[44]Denis, K. L. & Speidel, T. M. Comparison of three methods of profile change prediction in the adult orthodontic patient. *Am. J. Orthod. Dentofacial Orthop.* **92**, 396–402 (1987).

[45]Faysal Talass, M., Tollaae, L. & Baker, R. C. Soft-tissue profile changes resulting from retraction of maxillary incisors. *Am. J. Orthod. Dentofacial Orthop.* **91**, 385–394 (1987).

[46]Wilkinson, C. M., Motwani, M. & Chiang, E. The relationship between the soft tissues and

the skeletal detail of the mouth. *J. Forensic Sci.* **48**, 728–732 (2003).
[47]Subtelny, J. D. A longitudinal study of soft tissue facial structures and their profile characteristics, defined in relation to underlying skeletal structures. *Am. J. Orthod.* **45**, 481–507 (1959).
[48]Stephan, C. N. & Henneberg, M. Predicting mouth width from inter-canine width--a75% rule. *J. Forensic Sci.* **48**, 725–727 (2003).
[49]Balueva, T. S. & Veselovskaya, E. V. New developments in facial reconstruction. *Archaeology, Ethnology and Anthropology of Eurasia* **1**, 143–150 (2004).
[50]Stephan, C. N. & Murphy, S. J. Mouth width prediction in craniofacial identification: cadaver tests of four recent methods, including two techniques for edentulous skulls. *J. Forensic Odontostomatol.* **26**, 2–7 (2008).
[51]Guyomarch, P. & Stephan, C. N. The validity of ear prediction guidelines used in facial approximation. *Journal of Forensic Sciences* **57**, 1427–1441 (2012).
[52]Renwick, N. *Ear Lobe Morphology and Its Relationship to the Mastoid Process. AXIS: The Online Journal of CAHID, 12. Centre for Anatomy and Human Identification (CAHID)*. (University of Dundee, Dundee, 2012).
[53]Fourie, Z., Damstra, J., Gerrits, P. O. & Ren, Y. Accuracy and reliability of facial soft tissue depth measurements using cone beam computer tomography. *Forensic Sci. Int.* **199**, 9– 14 (2010).
[54]Hwang, H.-S. *et al.* Facial soft tissue thickness database for craniofacial reconstruction in Korean adults. *J. Forensic Sci.* **57**, 1442–1447 (2012).
[55]Wilkinson, C. *Forensic Facial Reconstruction*. (Cambridge University Press, Cambridge, England, 2004).
[56]Wilkinson, C. & Rynn, C. *Craniofacial Identification*. (Cambridge University Press, Cambridge, England, 2012).
[57]Stephan, C. N. CRANIOFACIALidentification. www.craniofacialidentification.com (2023).
[58]El-Mehallawi, I. H. & Soliman, E. M. Ultrasonic assessment of facial soft tissue thicknesses in adult Egyptians. *Forensic Sci. Int.* **117**, 99–107 (2001).

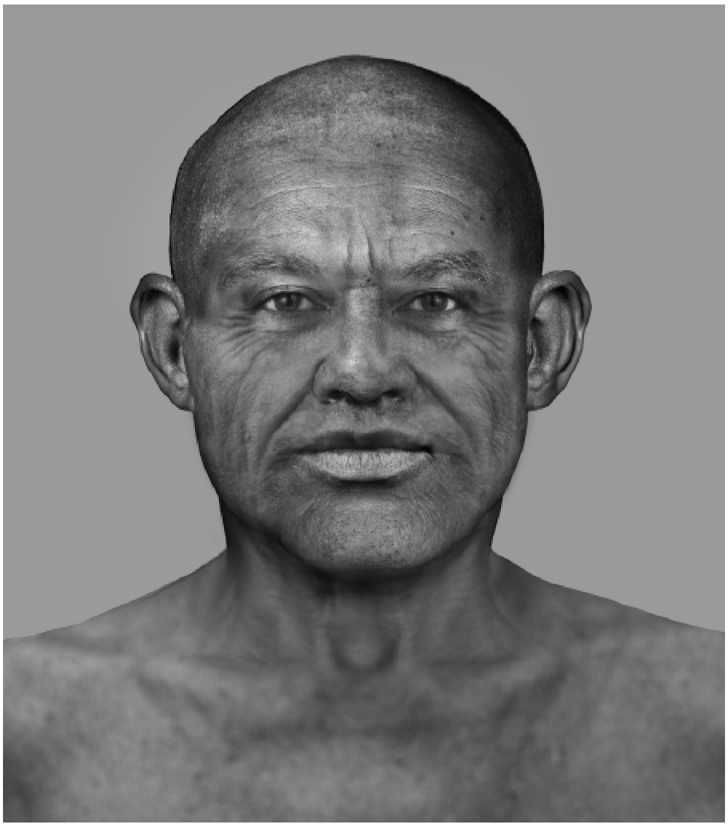

Figure S3.1. Facial reconstruction of the Nuwayrat individual (C. Wilkinson, Face Lab).

SI 4. Genetic ancestry modelling and admixture dating

*Corresponding author: adelinemorez@gmail.com (A. M. J.)

Modelling genetic ancestry with *qpAdm*

For all *qpAdm* modelling in this study, we estimated ancestry proportions as a mixture of a set of left (source) rotating populations differentially related to a set of right (outgroup) populations using ADMIXTOOLS2[1] qpadm_rotate() with the option maxmiss = 0.1, removing genotypes missing in >10% of populations. We restricted the analysis to genomes with both transitions and transversions (half/plus UDG-treated libraries or single-stranded libraries called using SequenceTools[2] --singleStrandMode), removing CpG sites, to increase the robustness of the models and avoid any potential biases introduced by postmortem molecular damage. We only considered models ≤ 3 sources. The following fixed outgroup set was used, restricted to genomes ≥ 2X: *Ju_hoan_North.DG, Ethiopia_4500BP.SG, Latvia_HG_UDG, USA_Ancient_Beringian.SG, Vanuatu_400BP_UDG, Japan_HG_Jomon_UDG, China_NEastAsia_Coastal_EN_UDG.* This analysis was conducted on the '1240K' dataset. These parameters are always true unless otherwise stated.

We ranked the non-rejected models (p > 0.05) first based on the number of source populations, where a model with one source population was considered to be more parsimonious than a model with two sources, and two sources considered more parsimonious than three sources. Then, if several models with the same number of sources are not rejected, we considered the p-value, given that the number of SNPs in the rotating models is nearly equal (10% missing), and used a p-value threshold of 0.05. When multiple models for a given rank (number of sources) had p > 0.05, we reported all these possibilities.

Distal qpAdm modelling

We first modelled Nuwayrat, Middle Neolithic Morocco, Bronze Age Anatolia and Bronze Age Levant (merged as one population or divided by archaeological site) as a combination of divergent 'distal' Epipalaeolithic/Neolithic populations from North Africa and West Asia (*Morocco_Epipaleolithic, Anatolia_Neolithic, Levant_Neolithic, Zagros_Neolithic, Caucasus_Neolithic*). Both *Caucasus_Neolithic* and *Zagros_Neolithic* were added despite their similarity because 1) the absence of *Zagros_Neolithic* resulted in no model passing p > 0.05 for NUE001 and 2) the absence of *Caucasus_Neolithic* resulted in no model passing p

> 0.05 for *Anatolia_BA*. Thus, both populations are necessary to effectively model the current set of target populations within the same framework. We used these populations instead of the more ancient ones associated with Palaeolithic culture due to their higher genome coverage, to avoid losing the power to discriminate between models. This analysis was carried out on 433,280-558,848 SNPs.

Only one three-source model fits the Nuwayrat genome, with 67.3 ± 3.7% Neolithic Levantine, 18.4 ± 3.1% Epipaleolithic Morocco and 14.3 ± 4.6% Neolithic Zagros ancestry (p = 0.07). The Nuwayrat genome is genetically similar to Middle Neolithic Morocco, with the exception that the latter shows an absence of the Zagros component. The proportion of Zagros component (or Caucasus component, also similar to Zagros Neolithic[4]) increases eastward (in the Levant and Anatolia), while the Epipaleolithic Morocco ancestry disappears (Extended Data Fig. 6, Supplementary Data Table 5).

Full qpAdm modelling

Bronze Age groups

We further modelled the ancestry of the Nuwayrat genome, as well as the two contemporary Bronze Age Anatolian and Levantine groups by including an extensive set of populations from Neolithic and Chalcolithic North Africa and West Asia as potential sources (*Morocco_EN_ktg, Morocco_MN, Anatolia_Neolithic, Anatolia_Chalcolithic, Levant_Neolithic, Levant_Chalcolithic, Zagros_Neolithic, Zagros_Chalcolithic, Mesopotamia_Neolithic, Caucasus_Neolithic, Caucasus_Chalcolithic*) as well as two Neolithic European groups: *Spain_EN* and *Greece_Neolithic*. In this model, we decided to keep the distal populations rotating between the left and right groups, given that, in the absence of older samples from Egypt, we can detect any admixture event predating the Nuwayrat individual. Given that no working hypothesis exists regarding the timing and source of an admixture event in the ancient Egyptians, this set-up was chosen to maximise the chances of finding the true sources of the admixture. This analysis was conducted over 474,731-578,969 SNPs.

No one-source model fit the Nuwayrat genome at p > 0.05 (maximum p-value observed = 2.39×10^{-6} for a model with *Morocco_MN* as a single source). Instead, a single two-source model met the significance criteria (p = 0.12). It estimates 77.6 ± 3.8% ancestry most closely related to the genomes from 4,780-4,230 BCE Middle Neolithic Morocco, and 22.4 ± 3.8% from the 9,000-8,000 BCE Neolithic Mesopotamia. The second best model included the

Middle Neolithic Morocco and Chalcolithic Zagros-related ancestry but can be rejected (p = 0.01). Both Neolithic Mesopotamia and Chalcolithic Zagros derive from similar ancestries (Extended Data Fig. 5)[3–5].

We also explored three-source models that met the significance criteria. The best-fitting three-source model includes 75.3 ± 6.4% Middle Neolithic Moroccan, 23.6 ± 5.3% Neolithic Mesopotamian, and 1.1 ± 8.7% Chalcolithic Levantine ancestries (p = 0.11, Supplementary Data Table 6), which replicates the Neolithic Mesopotamian-like signal in the best-fitting two-source model detailed above. This alternative model corroborates a scenario where the West Asian-related ancestry detected in the Nuwayrat individual is still most directly derived from Neolithic Mesopotamia and not from the two sources from which it derives, namely 1) Neolithic Levantine and 2) Caucasus/Zagros ancestries, which are related[4,5]. It is also worth noting that Neolithic Mesopotamia can be rejected as a single source for Nuwayrat's genetic ancestry ($p = 6.49 \times 10^{-75}$). Considering the two three-source alternative models, we cannot rule out the presence of additional Neolithic or Chalcolithic Levantine ancestries beyond what has already been identified in Neolithic Mesopotamia and Middle Neolithic Morocco (Supplementary Data Table 6), but these two models estimate a wide standard error range for the estimate of these Levantine ancestries - virtually resulting to a low or null proportion. Moreover, these two models are less probable than the favoured two-sources model (p = 0.11 and p = 0.07, respectively, against p = 0.12).

First, *qpAdm* modelling shows that the majority of the genetic ancestry in Nuwayrat derives from a North African Neolithic source which itself derives from a combination of local Palaeolithic/Epipalaeolithic ancestry, and Levant Neolithic/Chalcolithic ancestry that arrived at least 2,000 years prior the Nuwayrat individual life in northwestern Africa[6]. Second, it suggests that the significant presence of ancestry ultimately derived from the eastern Fertile Crescent, most similar to that of Neolithic Mesopotamia. This result is consistent with the previous distal model (Extended Data Fig. 6), but clarifies that the Zagros Neolithic-related ancestry found in Nuwayrat is derived from a source mostly similar to Mesopotamia Neolithic.

The presence of an eastern Fertile Crescent ancestry seemingly best represented by Mesopotamia Neolithic is surprising given the age difference between them and the Nuwayrat individual (~4,500 years apart). Zagros Chalcolithic genomes are from populations living near Mesopotamia but closer in time to the Nuwayrat individual and are represented by two archaeological sites, Tepe Hissar (near the southern shore of the Caspian Sea)[4,7] and Seh

Gabi (Zagros mountains)[3]. The individuals from these two sites show some level of genetic differentiation (Extended Data Fig. 5). Thus, we tested whether one of these two Zagros Chalcolithic sites could be a more proximal source for the eastern Fertile Crescent-derived component. We computed the above-mentioned *qpAdm* models, replacing *Zagros_Chalcolithic* with either one of the two sites. When the 4,848-3,792 BCE individuals from Seh Gabi are used, the best model following our criteria is 74.4 ± 4.1% *Morocco_MN* and 25.6 ± 4.1% *Mesopotamia_Neolithic* (p = 0.08, Supplementary Data Table 6). The best three-way model consists of 0.5 ± 5.6% *Levant_Chalcolithic*, 27.6 ± 5.6% *Mesopotamia_Neolithic* and 71.9 ± 6.4% *Morocco_MN* (p = 0.10). The previous best-fit two-source model is still favoured with this model, keeping Neolithic Mesopotamia as a more likely source than Seh Gabi. Considering the best-ranking three-source model, a similar model is conserved, with a low proportion of Chalcolithic Levant ancestry, consistent with 0%. When the 3,705-1,985 BCE individuals from Tepe Hissar are used to represent Chalcolithic individuals from Zagros, no model passes the selection threshold, similar to when both Tepe Hissar and Seh Gabi are added to the model as separate sources (Supplementary Data Table 6).

To further test the presence of an eastern Fertile Crescent-related ancestry in the Nuwayrat genomes, we generated the *F4-statistics* (Methods) testing which population breaks the assumption that Middle Neolithic Morocco and the Nuwayrat individual form a clade as f_4*(NUE001, Morocco_MN; Seh Gabi or Tepe Hissar, Ju hoan)*. Seh Gabi and Tepe Hissar both show increased allele sharing with the Nuwayrat individual ($f_4 > 0$; Z-scores = 3.07 and 1.91, respectively), returning an f_4-value significantly greater than 0 only when Seh Gabi is used, and lower but similar to when *Mesopotamia_Neolithic* is used as the potential admixing population (Fig. 2c).

Models of ancestry such as the *qpAdm* framework are most principled when sources predate the target individual whose ancestry is being modelled. The above-mentioned models only include sources that predate Nuwayrat. However, to further understand the Mesopotamian-related contribution to Nuwayrat, we added Bronze Age Levantine genomes as potential sources, which are contemporary to Nuwayrat and geographically proximate. None of the rotating models pass the significance threshold of p > 0.05. Noteworthy, the model with the highest p-value is 76.1 ± 3.8% Middle Neolithic Morocco and 23.9 ± 3.8% Neolithic Mesopotamia (p = 0.02), while a model fitting Middle Neolithic Morocco (71.3 ± 5.2%) and

Bronze Age Levant (28.7 ± 5.2%) returns a lower p-value (p = 0.01), although similar. The range of hypotheses that are consistent with the data thus includes that 1) the eastern Fertile-Crescent-related ancestry in Nuwayrat was due to processes that also impacted the Bronze Age Levant, 2) direct gene flow from the Bronze Age Levant spread the eastern Fertile Crescent ancestry into Egypt, or 3) limited statistical power does not allow to discriminate these two related sources.

Then, we also tested whether the constant best-fit of the Middle Neolithic Morocco in the *qpAdm* models might be caused by overfitting, since Middle Neolithic Morocco genomes are made of two main sources: Epipaleolithic Morocco and Neolithic Levant (Extended Data Fig. 6, Supplementary Data Table 5). The consequences of a potential overfitting could be twofold: 1) any significant presence of more direct Levantine ancestry might be best-modelled by Middle Neolithic Morocco, and 2) wrongly attribute *Mesopotamia_Neolithic* as the best-fit for the source of the eastern Fertile Crescent-related genetic component. We computed the following rotating models: *Morocco_EN_ktg, Morocco_Epipaleolithic, Anatolia_Neolithic, Anatolia_Chalcolithic, Levant_Neolithic, Levant_Chalcolithic, Zagros_Neolithic, Zagros_Chalcolithic, Mesopotamia_Neolithic, Caucasus_Neolithic, Caucasus_Chalcolithic*, *Spain_EN* and *Greece_Neolithic*, replacing Middle Neolithic Morocco by Epipalaeolithic Morocco[6], letting Epipaleolithic Morocco and Levantine populations fit freely the ancestry attributed to Middle Neolithic Morocco. None of the models could pass the selection criteria. We interpret this result as Middle Neolithic Morocco being the closest reference to the source for the North African genetic component, within the current available genomes included in this analysis. Additionally, including the Bronze Age Levant resulted in no model passing the significance threshold. This confirms that the Levantine-related ancestry present in the Nuwayrat genome (Extended Data Figure 6) is, at least in part, more directly similar to that of the Middle Neolithic Morocco than any other sources currently available. As a consequence, in the *qpAdm* models, the selection of Middle Neolithic Morocco is likely not contributing to a significant overfitting of the models, which limits its influence on the Neolithic Mesopotamia selected as the best source among the current population references.

All modelled Levantine Bronze Age groups trace 18.7-79.8% of their ancestry to previous Neolithic/Chalcolithic Levantine groups (except Megiddo and Yehud which could not be successfully modelled; Supplementary Data Table 6). If Nuwayrat received Neolithic Mesopotamian ancestry from the Bronze Age Levant, we should detect an additional

Neolithic/Chalcolithic Levant ancestry. However, in the two-source model, which is preferred, these ancestries are always at $p < 0.05$. In the three-way models, some models with these Neolithic/Chalcolithic Levantine ancestries pass the selection criteria but with either ancestry proportions overlapping 0% or with a lower p-value than when the model includes Neolithic Mesopotamia. Thus, as a most parsimonious explanation, we conclude that Neolithic Mesopotamia ancestry in Nuwayrat is not derived from that in Bronze Age Levant. We also detect ancestry from Neolithic Mesopotamia at three Levantine archaeological sites (Ebla, Baq'ah, Ashkelon) — considering the best-fit models —, at proportions (41.8-54.8%) exceeding that in the Nuwayrat genome. Altogether, the results confirm that the Mesopotamian Neolithic-related ancestry in some Bronze Age Levantines and Nuwayrat are likely distinct.

The best-fit model for Anatolia Bronze Age is 15.3 ± 3.2% Levant Neolithic and 84.7 ± 3.2% Caucasus Neolithic ($p = 0.15$; Supplementary Data Table 6), consistent with previous results considering that Caucasus Neolithic genomes were previously modelled as a mixture of Caucasus Palaeolithic and Anatolia Neolithic-related ancestries[4,8].

Overall, the ancestry of the Nuwayrat individual derives ~80% from previous local North African populations. The remaining ~20% ultimately derive from eastern Fertile Crescent populations. Among the current reference populations, the genomes most similar to the source of the eastern Fertile Crescent component can be attributed to Neolithic Mesopotamians. However, this ancestry also contributes to some Bronze Age Levant groups. The genetic similarity between these populations limits strong conclusions on the direct source of the eastern Fertile Crescent genetic ancestry and can be explained by a multitude of non-mutually exclusive demographic scenarios. The best fit of Mesopotamia Neolithic could be explained either by an admixture event ultimately involving North African-related and Mesopotamia-related populations starting much earlier, somewhere between 9,000 and 5,000 BCE. It could also be explained by a more recent admixture event, after 5,000 BCE, from a population most similar to Neolithic Mesopotamia or equally similar to Neolithic Mesopotamia and Bronze Age Levant.

Third Intermediate period individuals

We estimated ancestry proportions of the two Third Intermediate period Egyptians[9] using North African and West Asian populations who lived between the Old Kingdom and Third Intermediate Period (*NUE001, Anatolia_BA, Levant_BA, Iran_BA, Caucasus_BA*), as well as

a Bronze Age Greek population (*Greece_Minoan*). We also added *Morocco_MN* and *Mesopotamia_N* to test whether the Third Intermediate period Egyptians share a closer ancestry with Nuwayrat or a source more related to one of these two ancestries present in Nuwayrat. This analysis was conducted on 290,269 SNPs.

We reject all one-source models, including one with 100% continuity from Nuwayrat to the Third Intermediate Period ($p = 3.00 \times 10^{-7}$). The best-fit two-source model estimates 64.5 ± 5.6% Bronze Age Levant and 35.5 ± 5.6% Middle Neolithic Morocco ancestry (p = 0.32). An additional two-source model passes rejection thresholds but with lower p-values: Nuwayrat (44.5 ± 5.7%) and Bronze Age Levant (55.5 ± 5.7%) (p = 0.06). Two three-source models also pass the rejection threshold: 1) Bronze Age Levant (46.0 ± 28.0 %), Middle Neolithic Morocco (35.8 ± 5.8%), and Neolithic Mesopotamia (18.2 ± 26.0 %) (p = 0.24), and 2) Bronze Age Levant (46.0 ± 28.0%), Middle Neolithic Morocco (34.6 ± 5.6%), and Bronze Age Caucasus (0.9 ± 7.8%) (p = 0.21).

These models suggest that part of the Third Intermediate period individuals' ancestry derived from a population related to the Nuwayrat individual, either represented by NUE001 or the genomes from Middle Neolithic Morocco. Most of the Third Intermediate period ancestry derives from Bronze Age Levantine-related groups. Considering the two three-sources models, a third ancestry either represented by the Neolithic Mesopotamian or the Bronze Age Caucasus can be detected, but at levels consistent with 0%.

Given that Middle Neolithic Morocco and Neolithic Mesopotamia should capture NUE001 ancestry, and so be redundant in this model, we generated another rotating *qpAdm* approach, removing the Middle Neolithic Morocco and Neolithic Mesopotamia from the analysis (290,261 SNPs). This results in no one-, two- or three-source model passing the significance criteria of $p > 0.05$, suggesting that *Morocco_MN* is a necessary source for modelling the ancestry of the Third Intermediate Period Egyptian and that this ancestry was likely widespread in Egypt but potentially in varying proportion, leading to Middle Neolithic Morocco to be the most favoured source for the Third Intermediate Period ancestry modelling.

Ancient East Africans

Pastoralism in East Africa is associated with the spread of an Eurasian-like genetic component that comes from North African or Levantine populations[10,11]. We investigated whether the Nuwayrat genome is a better fit for the source of the Eurasian-like component

than the Chalcolithic Levant genomes. We estimated ancestry proportion in ancient East African genomes associated with Late Stone Age, Neolithic Pastoral and Iron Age cultures (Supplementary Data Table 3), using the set of rotating left groups: *NUE001, Levant_Chalcolithic, Ethiopia_4500BP.SG, Dinka.DG, Congo_Kindoki_Protohistoric, South_Africa_2200BP.SG.* For this model, the following fixed right groups were used: *Chimp.REF, Latvia_HG_UDG, USA_Ancient_Beringian.SG, Vanuatu_400BP_UDG, Japan_HG_Jomon_UDG, China_NEastAsia_Coastal_EN_UDG.* The one-, two-, or three-sources model passing rejection criteria at $p > 0.05$ are reported in Supplementary Data Table 12.

Most pastoral-related groups could be modelled with the Nuwayrat genome as a source (7.2 - 52.5%, Supplementary Data Table 12). The remaining ancestries are consistent with previous reports[10–13]. In a few cases, genomes from Chalcolithic Levant were favoured instead of NUE001 (1.0 - 46.4%), but the presence of this ancestry does not appear correlated to any particular cultural group.

Dating admixture in the Nuwayrat genome

We used DATES[14] to estimate admixture time between the Middle Neolithic Morocco genomes (source 1) and a set of eastern Fertile Crescent sources (source 2) in the Nuwayrat genome (target). We first used Neolithic genomes from Mesopotamia, the Zagros region or the Caucasus as a potential source for the eastern Fertile Crescent admixture, using the pseudo-haploid dataset. None of the DATES models passed the recommended validation threshold (nrmsd < 0.7 and Z-score > 2)[14], meaning it failed to accurately fit the ancestry covariance curve decay to the data (Supplementary Data Table 11). No model passed the validation threshold when we reiterated the analysis of the imputed genomes to improve the statistical power by increasing the number of SNPs (Supplementary Data Table 11). This was also the case when we expanded source 2 to be a Chalcolithic or Bronze Age population from the Levant, Zagros or Caucasus.

One likely explanation is the lack of statistical power to discriminate closely related sources in a single target genome. Alternatively, multiple events or gene flow spanning hundreds or thousands of years could result in such distribution of ancestry covariance within the Nuwayrat genome.

Basal Eurasian ancestry estimation

The contribution from the hypothetical Basal Eurasian lineage was estimated following the Lazaridis *et al.*[3] approach. We estimated the proportion of Basal Eurasian admixture (α) in North African and West Asian populations dated from the Palaeolithic to Bronze Age using the f_4*-ratio* on the form *f_4(Kostenki14, Ust'Ishim/CHS ; Test, Loschbour)/f_4(Kostenki14, Ust'Ishim/CHS ; Mota, Loschbour)* and on the form *f_4(Kostenki14, Ust'Ishim/CHS ; Test, Loschbour)/f_4(Kostenki14, Ust'Ishim/CHS ; ESN, Loschbour)*. The f_4*-ratio* was computed with the *admixr* R package[15] including all individuals from each group and restricting the analysis to transversions only.

The Nuwayrat genome has among the highest proportion of 'Basal Eurasian' ancestry observed among ancient DNA genomes (50.8-70.5%), most similar to genomes from Palaeolithic and Neolithic Morocco and Neolithic to Bronze Age Zagros (Figure S4.1), which is consistent with the best-fit *qpAdm* model.

References

[1]Maier, R. *et al.* On the limits of fitting complex models of population history to f-statistics. *Elife* **12**, (2023).

[2]Schiffels, S. *SequenceTool*. (2022).

[3]Lazaridis, I. *et al.* Genomic insights into the origin of farming in the ancient Near East. *Nature* **536**, 419–424 (2016).

[4]Lazaridis, I. *et al.* Ancient DNA from Mesopotamia suggests distinct Pre-Pottery and Pottery Neolithic migrations into Anatolia. *Science* **377**, 982–987 (2022).

[5]Altınışık, N. E. *et al.* A genomic snapshot of demographic and cultural dynamism in Upper Mesopotamia during the Neolithic Transition. *Sci Adv* **8**, eabo3609 (2022).

[6]Simões, L. G. *et al.* Northwest African Neolithic initiated by migrants from Iberia and Levant. *Nature* **618**, 550–556 (2023).

[7]Narasimhan, V. M. *et al.* The formation of human populations in South and Central Asia. *Science* **365**, eaat7487 (2019).

[8]Skourtanioti, E. *et al.* Genomic History of Neolithic to Bronze Age Anatolia, Northern Levant, and Southern Caucasus. *Cell* **181**, 1158–1175.e28 (2020).

[9]Schuenemann, V. J. *et al.* Ancient Egyptian mummy genomes suggest an increase of Sub-

Saharan African ancestry in post-Roman periods. *Nat. Commun.* **8**, 15694 (2017).

[10]Wang, K. *et al.* Ancient genomes reveal complex patterns of population movement, interaction, and replacement in sub-Saharan Africa. *Science Advances* **6**, eaaz0183 (2020).

[11]Prendergast, M. E. *et al.* Ancient DNA reveals a multistep spread of the first herders into sub-Saharan Africa. *Science* **365**, (2019).

[12]Lipson, M. *et al.* Ancient DNA and deep population structure in sub-Saharan African foragers. *Nature* **603**, 290–296 (2022).

[13]Lipson, M. *et al.* Ancient West African foragers in the context of African population history. *Nature* **577**, 665–670 (2020).

[14]Chintalapati, M., Patterson, N. & Moorjani, P. The spatiotemporal patterns of major human admixture events during the European Holocene. *Elife* **11**, (2022).

[15]Petr, M., Vernot, B. & Kelso, J. admixr—R package for reproducible analyses using ADMIXTOOLS. *Bioinformatics* **35**, 3194–3195 (2019).

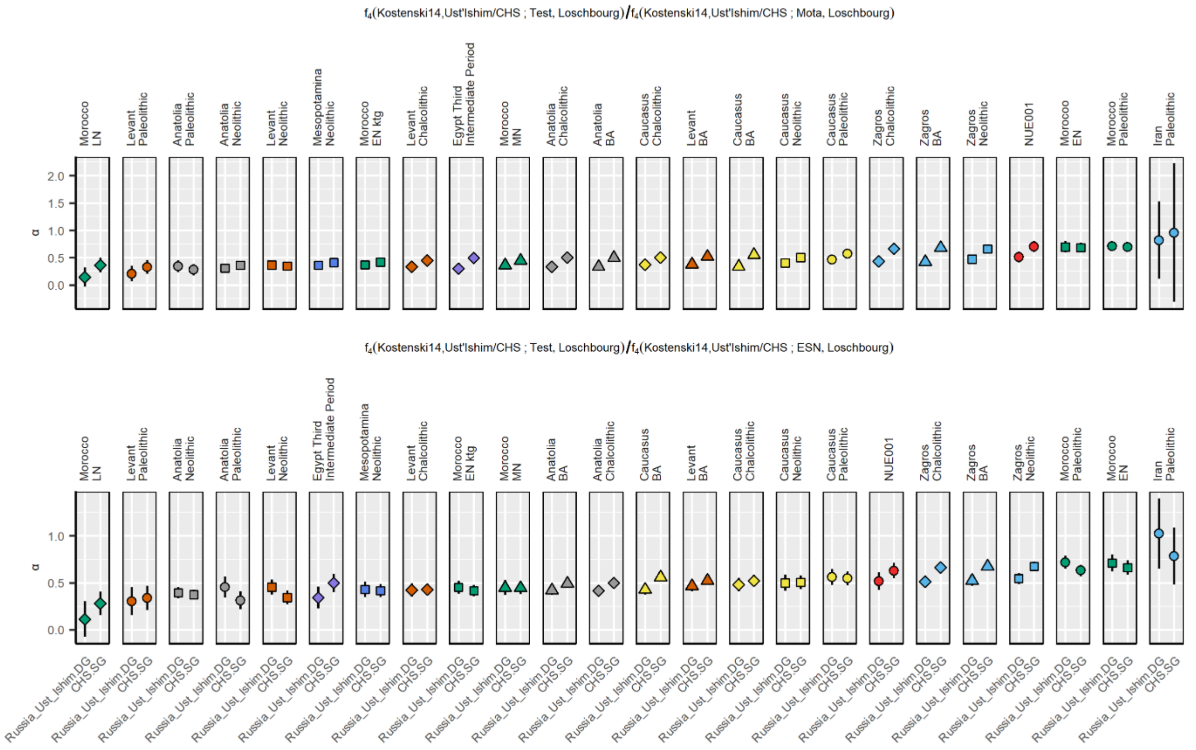

Figure S4.1. Basal Eurasian proportion in ancient West Asian and North African groups, estimated using *f_4-ratio*. CHS, present-day Southern Han Chinese genomes; ESN, present-day West African Esan genomes. This analysis was conducted over 276,317 SNPs. Symbols represent f_4-ratio coefficient (∝) ± 1 SE (error bars).

SI 5. Stable isotopes analyses

*Corresponding authors: MadgwickRD3@cardiff.ac.uk (R. M.) and HoltE@cardiff.ac.uk (E. H.)

Isotope Analysis – A Brief Overview

Elemental isotopes are incorporated into skeletal structures through the consumption of foods, beverages, and drinking water, reflecting an individual's dietary environment and making isotopes useful for reconstructing past diets and origins. Drivers of isotope variation are complex and varied; they have been simplified here to provide context for the current study.

For carbon, the isotope values ($\delta^{13}C$) of plants are influenced by landscape carbon baselines, different photosynthetic pathways (e.g. C_3 vs. C_4), and sea-spray (if present). Because plants form the basis of food chains, the carbon isotopes in plants get progressively incorporated into higher level consumers, increasing in value slightly with each consumer[1]. Nitrogen isotope values ($\delta^{15}N$) principally indicate the trophic level of the individual, with the lowest values representing terrestrial herbivores and the highest values representing marine carnivores[1]. Individuals with marine diets tend to have higher nitrogen isotope values due to the elongated food chains in marine ecosystems, although nitrogen values can also be affected by environmental factors. For example, an increase in salinity in the growing environment will result in an increase in nitrogen isotope values in plants and the animals that eat them[2].

Carbon and nitrogen isotope values are generally obtained by analysing collagen extracted from tissues such as bone, dentine, skin, and hair. Isotope values from collagen extracted from various skeletal elements reflect variable spans of time in an individual's life based on when the element is first formed and the element's remodelling rate thereafter. For example, human ribs remodel fairly rapidly and are thought to represent a dietary average of approximately five years before death[3,4]. Dentine forms during childhood and only minimally remodels during an individual's lifetime; collagen extracted from dentine was therefore examined in this study to investigate the individual's childhood diet.

Geographic origins can be explored using isotope ratios from strontium and oxygen, among others. Variation in strontium ($^{87}Sr/^{86}Sr$) ratios reflects primarily the different underlying

bedrocks where the food an individual consumed was sourced, with older lithologies providing more radiogenic values[5,6]. Variation in oxygen ($\delta^{18}O$) values is principally determined by climate, with higher values indicating a hotter, drier climate. Strontium and oxygen are analysed from dental enamel, a highly mineralized tissue that does not remodel and therefore provides a signal for the time during childhood when it was formed.

Methods

A single lower left second molar from an adult male was sampled for dental collagen extraction and enamel. The enamel sampled forms at approximately 5 to 9 years of age, while the tooth roots form from about 8 to 13 years of age[7].

Collagen

Collagen was extracted from the specimen's dental root for carbon ($\delta^{13}C$) and nitrogen ($\delta^{15}N$) isotope analysis. Collagen extraction was undertaken at Cardiff University BioArchaeology labs following a modified Longin method[8,9]. The surface of the root was lightly abraded with a diamond-coated burr, then a sample was extracted using a diamond wheel. Approximately half of the cervical part of one root was taken, with the section weighing 0.12 grams. The sample was demineralized in 0.5M HCl until soft, then rinsed with deionized water and gelatinized in pH3 H_2O on a hot block at 75°C. After 48 hours, the sample was removed from the hot block, filtered using an Ezee™ filter, transferred to a polypropylene test tube, and frozen overnight. The sample was then lyophilized, leaving dry gelatinized collagen. Analysis was undertaken using continuous flow mass spectrometry at Cardiff University Stable Isotope Facility using a Flash 1112 series elemental analyser coupled to a ThermoFinnigan Delta V Advantage using protocols described in Scorrer et al.[10]. The analytical precision (one standard deviation) of the gelatine and caffeine standards was 0.08 and 0.07 for $\delta^{13}C$ and $\delta^{15}N$, respectively, over the analytical session in which this sample was analysed.

Enamel

Enamel was extracted from the region of the Cementum Enamel Junction (CEJ), which in the second molar develops between 5 and 9 years old[7]. The surface of the enamel was first abraded using a diamond coated burr. Then, a slice of enamel 3.6 mm in width was extracted using a precision dental drill, and all dentine was abraded away. Two small fragments of enamel from the same section were crushed using an agate mortar and pestle: one sample

was ultrasonicated before being crushed, the other was not. A minimum of 3.0 mg of each crushed sample was weighed into a microcentrifuge tube for $\delta^{18}O$ analysis, and the isotope composition of the structural carbonate within the enamel was measured ($\delta^{18}O_{carbonate}$) at the Cardiff University Stable Isotope Facility. Samples were acidified for 5 minutes with >100% ortho-phosphoric acid at 70°C and analysed using a Thermo MAT253 dual inlet mass spectrometer coupled to a Kiel IV carbonate preparation device using protocols described in Scorrer et al.[10]. Both samples were run in duplicate. The resultant isotope values are reported as per mil ($^{18}O/^{16}O$) normalized to the VPDB scale using an in-house carbonate reference material (BCT63) calibrated against NBS19 certified reference material. The long-term reproducibility for $\delta^{18}O$ BCT63 is ±0.04 per mil and ± 0.03 for $\delta^{13}C$ (1σ). Oxygen carbonate values ($\delta^{18}O_{C\ VPDB}$) were converted to the SMOW scale using the conversion equation of Coplen[11] and to phosphate ($\delta^{18}O_{P\ VSMOW}$) using the formula from Chenery et al.[12].

The remaining enamel fragment was cleaned in an ultrasonic bath, rinsed, and dried. This clean enamel fragment weighing 56.29 mg was stored in a microcentrifuge tube and transferred to a clean working area (class 100, laminar flow) at Cardiff Earth Laboratory for Trace Element and Isotope Chemistry (CELTIC) for further sample preparation and $^{87}Sr/^{86}Sr$ isotope analysis. The sample was digested in 8M HNO_3 and heated overnight at 120°C. Strontium extraction used Sr Spec™ resin following a revised version of the protocol of Font et al.[13]. The sample was loaded into a resin column in 1 ml 8M HNO_3. Matrix elements (including calcium and traces of rubidium) were then eluted in several washes of 8M HNO_3 and the samples placed on a hotplate (120°C) overnight. This process was then repeated for a second pass to remove all remaining calcium. Once the purified sample was dry, the sample was re-dissolved in 2% HNO_3. The strontium isotope ratio was measured using a Thermo Fisher Scientific Neoma MC-ICP-MS/MS installed May 2024 at Cardiff University. Instrumental mass bias was corrected for using the exponential law and a normalization ratio of 8.375209 for $^{88}Sr/^{86}Sr$[14]. Residual krypton (Kr) and rubidium (^{87}Rb) interferences were monitored and corrected for using ^{84}Kr and ^{86}Kr ($^{83}Kr/^{84}Kr$ = 0.20175 and $^{83}Kr/^{86}Kr$ = 0.66474; without normalization) and ^{85}Rb ($^{85}Rb/^{87}Rb$ = 2.5926), respectively. Accuracy of the method was assessed by measurement of the EC-5 coral standard during the analytical session, which gave an $^{87}Sr/^{86}Sr$ value of 0.709171 ± 0.000016 (2σ, n=14), consistent with the expected value of approximately sea water (0.709180). Data is also corrected against a NIST SRM 987 value of 0.710248[15]. The procedural blank was less than 75pg of Sr, which is negligible relative to the amount of Sr in each sample.

Results

Carbon and Nitrogen

Carbon and nitrogen isotopes results are presented in Table S5.1. The processed collagen was subsampled and run in duplicate to ensure accuracy.

Table S5.1 $\delta^{13}C$ and $\delta^{15}N$ results for the Nuwayrat sample.

Individual	Element	Duplicate	$\delta^{15}N_{AIR}$ (‰)	$\delta^{13}C_{VPDB}$ (‰)	%N	%C	C:N
Col #50.33	Left LM2	A	12.3	-19.6	16.02	45.34	3.30
		B	12.3	-19.6	15.60	43.86	3.28

The duplicate subsamples of the extracted collagen had C:N atomic ratios of 3.30 and 3.28, indicating good collagen preservation[16] and also meeting the more stringent criteria set out by Guiry and Szpak[17].

Strontium and Oxygen

Strontium and oxygen isotope results are presented in Table S5.2. Subsamples of enamel for oxygen analysis were prepared using two different methods: one subsample (A) was not ultrasonicated before analysis (*NUS* in Table S5.2), following the standard protocol, and a second subsample (B) was ultrasonicated before analysis (*US* in Table S5.2) to test whether ultrasonication affected the results. Both subsamples were analysed in duplicate to ensure accuracy. The results for A1 and A2 were within one standard deviation of each other. B1 and B2 fell outside one standard deviation of each other and outside the one standard deviation of each of the A subsamples. While this suggests that ultrasonication may slightly alter the oxygen isotope composition of human tooth enamel, the results for all four samples are interpretatively equivalent.

Table S5.2 $^{87}Sr/^{86}Sr$ and $\delta^{18}O$ results for the Nuwayrat sample. "NUS" indicates that the sample was not ultrasonicated before analysis. "US" indicates that the sample was ultrasonicated before analysis.

Individual	$^{87}Sr/^{86}Sr$	2 SE Sr	O Duplicate	$\delta^{18}O_{C\ VPDB}$ (‰)	$\delta^{18}O_{P\ VSMOW}$ (‰)	$\delta^{13}C_{VPDB}$ (‰)
Col #50.33	0.707888	0.000006	A1 (NUS)	1.32	23.6	-13.00
			A2 (NUS)	1.30	23.6	-12.99
			B1 (US)	1.39	23.6	-13.20
			B2 (US)	1.47	23.7	-13.15

Discussion

Diet: Carbon and Nitrogen

It is necessary to consider the principal drivers of isotope value variation in an Egyptian context in discussing the results of this study. As discussed above, carbon isotopes inform on the photosynthetic pathway of the plants in the consumer's food chain (e.g. C_3 vs. C_4), as well as whether the consumer's diet was composed primarily of terrestrial or marine protein resources. Foods with a variety of $\delta^{13}C$ values were potentially available to the individual in this study. Typical Near Eastern domestic crops such as wheat and barley are C_3 plants, which exhibit low $\delta^{13}C$ values (~-26 ‰). Millet and sorghum were potentially available to the ancient Egyptians[18], and as C_4 plants, they have much higher $\delta^{13}C$ values (~-12 ‰). The consumption of imported marine resources was possible given existing trade routes[19], and marine plants' values typically fall between C_3 and C_4 terrestrial plants (~-19 ‰)[1]. Terrestrial ecosystems have relatively short food chains compared to marine ecosystems, resulting in terrestrial diets having lower $\delta^{13}C$ values than marine diets. A $\delta^{13}C$ value of -20.0 ‰ is typically considered the boundary for a completely terrestrial diet in a C_3 ecosystem[1,20].

The individual in this study had a mean $\delta^{13}C$ value of -19.6 ‰, consistent with an almost exclusively terrestrial C_3 diet. These results are similar to the results of previous isotope studies, which have found that a C_3 diet was remarkably standard throughout ancient Egypt until the Coptic Period[21]. A reliance on a C_3 diet in ancient Egypt is also indicated by figurative depictions, inscriptions, and the analysis of food remains in archaeological contexts[22–26].

Nitrogen isotopes principally reflect the trophic level of the consumer, with each successive

increase the food chain resulting in a ^{15}N enrichment of 3.0‰ to 6.0 ‰[1], although $\delta^{15}N$ values can also be affected by baseline environmental nitrogen (e.g., manured crops and salt marsh environments having higher baselines[2]). Meat consumption would lead to higher $\delta^{15}N$ values and was likely an important part of the diet for ancient Egyptians of many social classes based on figurative and zooarchaeological evidence[22,23,25,26]. Additionally, the consumption of animal products such as milk, cheese, and eggs can also lead to higher $\delta^{15}N$ values, and all played a role in ancient Egyptian diets[24]. As with $\delta^{13}C$, marine ecosystems exhibit higher $\delta^{15}N$ values than terrestrial ones due to their extended food chains; however, evidence for the consumption of marine fish in ancient Egypt has not been noted figuratively or zooarchaeologically[25]. Conversely, the consumption of freshwater Nile fish is represented in ancient Egyptian art[25] and has been noted zooarchaeologically at the Old Kingdom Workers' Town at Giza[22] and occasionally in food offerings for the dead[24]. Freshwater ecosystems also have extended food chains, although the freshwater environment has similarities with terrestrial baselines, resulting in freshwater $\delta^{15}N$ values that are intermediate between exclusively marine and exclusively terrestrial environments[1]. Individuals with higher proportions of freshwater fish in their diets will therefore have higher $\delta^{15}N$ values than those consuming sea spray affected terrestrial plants or low trophic level marine protein (e.g. marine plants, molluscs).

The mean $\delta^{15}N$ value of 12.3 ‰ of the analysed individual is higher than is generally expected for a terrestrial C_3 omnivore. Modern omnivorous Europeans and North Americans have been found to have values of ~9.5 ‰, and modern omnivorous Asians values of ~8.5‰[27,28]. However, a $\delta^{15}N$ value of 12.3 ‰ falls within the ranges previously measured in archaeological populations from ancient Egypt and Nubia[27,29,30]. There are many potential causes for elevated $\delta^{15}N$ values in terrestrial omnivores, and a likely possibility for the individual analysed in this report, as well as for individuals from ancient Egypt in general, is an increase in the environmental baseline of $\delta^{15}N$ due to aridity[31]. This hypothesis is supported by studies that have found elevated $\delta^{15}N$ values in herbivorous fauna from Egypt and Nubia, and even in some plants[21,27,29,30,32,33].

Another possible explanation for the individual's elevated $\delta^{15}N$ value is that the crops and animals that made up this individual's diet were grown or raised on manured fields, as has been suggested for several individuals dating to the predynastic period in Egypt[34]. The application of manure from different animals can have a substantial effect on the $\delta^{15}N$ values

of plants grown in the improved soil[35].

A third possible explanation is the consumption of aquatic resources such as freshwater fish. Nile perches have $\delta^{15}N$ values from +8‰ to +11 ‰, meaning that humans who consume large amounts of Nile perch would be expected to have $\delta^{15}N$ values of at least +13 ‰[32]. Sulfur isotope values ($\delta^{34}S$) have been used in some studies to distinguish high $\delta^{15}N$ values due to fish consumption from high $\delta^{15}N$ values due to other factors, a $\delta^{34}S$ value of approximately +10 ‰ being expected if a significant amount of Nile perch was consumed[34]; however, a $\delta^{34}S$ value is not available for the analysed individual.

A final possible cause of elevated $\delta^{15}N$ values is nutritional stress, which alters nitrogen metabolism. Although small increases in $\delta^{15}N$ have been noted in pregnant women[36], this situation would not apply to the analysed individual, who was a male. Larger increases in $\delta^{15}N$ can be caused by severe food stress/starvation[37]. However, given that elevated $\delta^{15}N$ values have been identified in ancient Egyptian and Nubian individuals from all periods, environmental or dietary explanations for the studied individual's elevated $\delta^{15}N$ value are more likely.

Provenancing: Strontium and Oxygen

The $^{87}Sr/^{86}Sr$ ratios from enamel represent the bioavailable strontium; that is, the strontium that can be incorporated into vegetation and subsequently into other food sources. The greatest contributing factor to bioavailable strontium is generally the local bedrock; however, near the Nile River, the majority of sediments are not local but have been transported to the area through seasonal flooding. A previous study has found that the mean $^{87}Sr/^{86}Sr$ values of tooth enamel from New Kingdom-period individuals from both Qurneh (Middle Egypt) and Memphis (Lower Egypt) are 0.70777 (sd 0.00017 and sd 0.00034 respectively)[38]. Further studies with larger sample sizes incorporating both human and animal specimens have found that Nile River $^{87}Sr/^{86}Sr$ values are remarkably consistent, with a mean of 0.70769±0.00003[39]. The $^{87}Sr/^{86}Sr$ value of the individual in this study is therefore consistent with a childhood spent near the Nile River.

Interpretations of mobility and origins are more robust when data from different isotope proxies are combined, rather than assessed in isolation. The individual in this study had a mean $\delta^{18}O$ value of 23.6 ‰, indicating quite a hot, dry climate. This value is consistent with oxygen values found by a previous study of human tooth enamel from ancient Egyptian

individuals from a variety of sites along the Nile dating to the predynastic through Coptic periods[40]. Although the value of the Old Kingdom individual analysed in the present study most closely matches the individuals from the Touzeau et al.[40] study dating to the Late Period through Greco-Roman period, the sample sizes in the Touzeau et al.[40] study are small, and the sampled individuals come from diverse sites. These individuals show a range of $\delta^{18}O$ values, including one individual from Middle Kingdom Deir el Medineh with a tooth enamel $\delta^{18}O$ value of 23.4 ‰[40], similar to the high $\delta^{18}O$ value of the individual in this study.

Conclusions

The existing isotope studies of individuals from ancient Egypt show a notable consistency over time and place. Carbon and nitrogen analyses suggest little dietary change until the Coptic period, and the results of strontium analyses are comparable throughout the Nile Valley. Oxygen is the exception. Oxygen analyses show a shift over time suggesting a progressively hotter, drier climate, although sample sizes are small and there is substantial variation within time periods. The results of all analysed isotopes for the individual in this study are consistent with a person who grew up in the Nile Valley eating a typical ancient Egyptian diet.

References

[1]Richards, M. P. Isotope analysis for diet studies. in *Archaeological Science* 125–144 (Cambridge University Press, 2019).

[2]Britton, K., Müldner, G. & Bell, M. Stable isotope evidence for salt-marsh grazing in the Bronze Age Severn Estuary, UK: implications for palaeodietary analysis at coastal sites. *J. Archaeol. Sci.* **35**, 2111–2118 (2008).

[3]Fahy, G. E., Deter, C., Pitfield, R., Miszkiewicz, J. J. & Mahoney, P. Bone deep: Variation in stable isotope ratios and histomorphometric measurements of bone remodelling within adult humans. *J. Archaeol. Sci.* **87**, 10–16 (2017).

[4]Cox, G. & Sealy, J. Investigating Identity and Life Histories: Isotopic Analysis and Historical Documentation of Slave Skeletons Found on the Cape Town Foreshore, South Africa. *Int J Hist Archaeol* **1**, 207–224 (1997).

[5]Holt, E., Evans, J. A. & Madgwick, R. Strontium (87Sr/86Sr) mapping: A critical review of methods and approaches. *Earth Sci. Rev.* **216**, 103593 (2021).

[6]Evans, J. A., Montgomery, J., Wildman, G. & Boulton, N. Spatial variations in biosphere

87Sr/86Sr in Britain. *J Geol Soc London* **167**, 1–4 (2010).
[7]AlQahtani, S. J., Hector, M. P. & Liversidge, H. M. Brief communication: The London atlas of human tooth development and eruption. *Am. J. Phys. Anthropol.* **142**, 481–490 (2010).
[8]Brown, T. A., Nelson, D. E., Vogel, J. S. & Southon, J. R. Improved collagen extraction by modified Longin method. *Radiocarbon* **30**, 171–177 (1988).
[9]Longin, R. New method of collagen extraction for radiocarbon dating. *Nature* **230**, 241–242 (1971).
[10]Scorrer, J. *et al.* Diversity aboard a Tudor warship: investigating the origins of the Mary Rose crew using multi-isotope analysis. *R. Soc. Open Sci.* **8**, 202106 (2021).
[11]Coplen, T. B. Normalization of oxygen and hydrogen isotope data. *Chem. Geol.* **72**, 293–297 (1988).
[12]Chenery, C. A., Pashley, V., Lamb, A. L., Sloane, H. J. & Evans, J. A. The oxygen isotope relationship between the phosphate and structural carbonate fractions of human bioapatite. *Rapid Commun. Mass Spectrom.* **26**, 309–319 (2012).
[13]Font, L., Nowell, G. M., Graham Pearson, D., Ottley, C. J. & Willis, S. G. Sr isotope analysis of bird feathers by TIMS: a tool to trace bird migration paths and breeding sites. *J. Anal. At. Spectrom.* **22**, 513 (2007).
[14]Nier, A. O. The isotopic constitution of strontium, barium, bismuth, thallium and mercury. *Phys. Rev.* **54**, 275–278 (1938).
[15]Avanzinelli, R., Conticelli, S. & Francalanci, L. High precision Sr, Nd, and Pb isotopic analyses using the new generation Thermal Ionisation Mass Spectrometer ThermoFinnigan Triton-Ti®. *Periodico di Mineralogia* **74**, 147–166 (2015).
[16]DeNiro, M. J. Postmortem preservation and alteration of in vivo bone collagen isotope ratios in relation to palaeodietary reconstruction. *Nature* **317**, 806–809 (1985).
[17]Guiry, E. J. & Szpak, P. Improved quality control criteria for stable carbon and nitrogen isotope measurements of ancient bone collagen. *J. Archaeol. Sci.* **132**, 105416 (2021).
[18]Brewer, D. J., Redford, D. B. & Redford, S. *Domestic Plants and Animals: The Egyptian Origins*. (Oxbow Books, Oxford, 2024).
[19]Mumford, G. Ras Budran and the Old Kingdom trade in Red Sea shells and other exotica. *British Museum Studies in Ancient Egypt and Sudan* **18**, 107–145 (2012).
[20]Richards, M. P., Fuller, B. T. & Molleson, T. I. Stable isotope palaeodietary study of humans and fauna from the multi-period (Iron Age, Viking and Late Medieval) site of Newark Bay, Orkney. *J. Archaeol. Sci.* **33**, 122–131 (2006).

[21]Touzeau, A. *et al.* Diet of ancient Egyptians inferred from stable isotope systematics. *J. Archaeol. Sci.* **46**, 114–124 (2014).
[22]Redding, R. W., Campana, D., Crabtree, P., Lev-Tov, S. D. & Choyke, J. Status and Diet at the Workers' Town. in *Anthropological Approaches to Zooarchaeology. Complexity, Colonialism, and Animal Transformations* 65–75 (Oxbow Books, Giza,Egypt; Cambridge, 2010).
[23]Alcock, J. P. *Food in the Ancient World*. (Bloomsbury Academic, London, England, 2005).
[24]Bresciani, E. *Food Culture in Ancient Egypt. in Food: A Culinary History*. (Columbia University Press, New York, 2013).
[25]Ikram, S. *Choice Cuts: Meat Production in Ancient Egypt*. (Peeters, Leuven, Belgium, 1995).
[26]Gilbert, A. S. Zooarchaeological Observations on the Slaughterhouse of Meketre. *J Egypt Archaeol* **74**, 69–89 (1988).
[27]Macko, S. A. *et al.* Documenting the diet in ancient human populations through stable isotope analysis of hair. *Philos. Trans. R. Soc. Lond. B Biol. Sci.* **354**, 65–76 (1999).
[28]Thompson, A. H. *et al.* Stable isotope analysis of modern human hair collected from Asia (China, India, Mongolia, and Pakistan). *Am. J. Phys. Anthropol.* **141**, 440–451 (2010).
[29]Thompson, A. H., Richards, M. P., Shortland, A. & Zakrzewski, S. R. Isotopic palaeodiet studies of Ancient Egyptian fauna and humans. *J. Archaeol. Sci.* **32**, 451–463 (2005).
[30]Thompson, A. H., Chaix, L. & Richards, M. P. Stable isotopes and diet at Ancient Kerma, Upper Nubia (Sudan). *J. Archaeol. Sci.* **35**, 376–387 (2008).
[31]Wu, Y., Wang, B. & Chen, D. Regional-scale patterns of δ13C and δ15N associated with multiple ecosystem functions along an aridity gradient in grassland ecosystems. *Plant Soil* **432**, 107–118 (2018).
[32]Iacumin, P., Bocherens, H., Mariotti, A. & Longinelli, A. An isotopic palaeoenvironmental study of human skeletal remains from the Nile Valley. *Palaeogeogr. Palaeoclimatol. Palaeoecol.* **126**, 15–30 (1996).
[33]Dupras, T. L. & Schwarcz, H. P. Strangers in a strange land: Stable isotope evidence for human migration in the dakhleh oasis, Egypt. *J. Archaeol. Sci.* **28**, 1199–1208 (2001).
[34]Poulallion, E. *et al.* High δ^{15}N values in Predynastic Egyptian archeological remains: A potential indicator for localised soil fertilisation practices in extreme conditions. *bioRxiv* (2024) doi:10.1101/2024.11.18.624066.
[35]Szpak, P. Complexities of nitrogen isotope biogeochemistry in plant-soil systems: implications for the study of ancient agricultural and animal management practices. *Front.*

Plant Sci. **5**, 288 (2014).

[36]Fuller, B. T. Nitrogen balance and δ15N: Why you're not what you eat during nutritional stress. *Rapid Communications in Mass Spectrometry* **19**, 2497–2506 (2005).

[37]Neuberger, F. M., Jopp, E., Graw, M., Püschel, K. & Grupe, G. Signs of malnutrition and starvation-Reconstruction of nutritional life histories by serial isotopic analyses of hair. *Forensic Sci Int* **226**, 22–32 (2013).

[38]Buzon, M. R. & Simonetti, A. Strontium isotope (87Sr/86Sr) variability in the Nile Valley: identifying residential mobility during ancient Egyptian and Nubian sociopolitical changes in the New Kingdom and Napatan periods. *Am. J. Phys. Anthropol.* **151**, 1–9 (2013).

[39]Stantis, C., Nowell, G. M., Prell, S. & Schutkowski, H. Animal proxies to characterize the strontium biosphere in the northeastern Nile Delta. *Bioarchaeology of the Near East* **13**, 1–13 (2019).

[40]Touzeau, A. *et al.* Egyptian mummies record increasing aridity in the Nile valley from 5500 to 1500yr before present. *Earth Planet. Sci. Lett.* **375**, 92–100 (2013).
