# Supplementary material for: Whole-genome ancestry of an Old Kingdom Egyptian
Source: Nature. 2025 Jul 2;644(8077):714–21. doi: 10.1038/s41586-025-09195-5 (PMC12367555; doi:10.1038/s41586-025-09195-5)
Supplement: Supplementary file 2 — Reporting Summary [file 41586_2025_9195_MOESM2_ESM.pdf]

nature portfolio

Corresponding author(s): Adeline Morez Jacobs, Pontus Skoglund, Linus Girdland Flink

Last updated by author(s): 20/03/2025

Reporting Summary

Nature Portfolio wishes to improve the reproducibility of the work that we publish. This form provides structure for consistency and transparency in reporting. For further information on Nature Portfolio policies, see our Editorial Policies and the Editorial Policy Checklist.

Statistics

For all statistical analyses, confirm that the following items are present in the figure legend, table legend, main text, or Methods section.

n/a	Confirmed	
☐	☒	The exact sample size (*n*) for each experimental group/condition, given as a discrete number and unit of measurement
☐	☒	A statement on whether measurements were taken from distinct samples or whether the same sample was measured repeatedly
☐	☒	The statistical test(s) used AND whether they are one- or two-sided<br>*Only common tests should be described solely by name; describe more complex techniques in the Methods section.*
☒	☐	A description of all covariates tested
☒	☐	A description of any assumptions or corrections, such as tests of normality and adjustment for multiple comparisons
☐	☒	A full description of the statistical parameters including central tendency (e.g. means) or other basic estimates (e.g. regression coefficient) AND variation (e.g. standard deviation) or associated estimates of uncertainty (e.g. confidence intervals)
☐	☒	For null hypothesis testing, the test statistic (e.g. *F*, *t*, *r*) with confidence intervals, effect sizes, degrees of freedom and *P* value noted<br>*Give P values as exact values whenever suitable.*
☒	☐	For Bayesian analysis, information on the choice of priors and Markov chain Monte Carlo settings
☒	☐	For hierarchical and complex designs, identification of the appropriate level for tests and full reporting of outcomes
☒	☐	Estimates of effect sizes (e.g. Cohen's *d*, Pearson's *r*), indicating how they were calculated

Our web collection on statistics for biologists contains articles on many of the points above.

Software and code

Policy information about availability of computer code

Data collection	Sequence demultiplexing and adapater removal: AdapterRemoval v2.3.1.
Data analysis	A full description of all software and respective packages used for data analysis can be found in the Method section and are publicly available.<br>Sequencing reads processing: nf-core/eager v2.3.3 pipeline; Genomic read mapping: Burrows-Wheller Aligner (BWA, v0.7.17); Duplicates removal: Dedup v0.12.8; Aligned reads filtering: SAMtools v1.9.2; Substitution distribution: MapDamage v2; Genome-wide contamination rate using the Conditional Substitution Rate: PMDtools v0.60; mitochondrial DNA-based contamination and consensus: Schmutzi (commit be61017); X-chromosome-based contamination in males: ANGSD v0.933; Reads count for molecular sexing: SAMtools v1.9.2; Sequencing runs merging: SAMtools v1.9.2; Pseudo-haploid SNP calling: SAMtools v1.9.2 and SequenceTools v1.5; mitochondrial haplogroup assignement: Haplogrep v3.2.1 and PhyloTree mtDNA tree build 17 (18 Feb 2016); Y-chromosome haplogroup assignement: pathPhynder and the International Society Of Genetic Genealogy (ISOGG v15.73); Genomic dataset management (including LD pruning and datasets merging): PLINK v. 1.9, EIGENSOFT 6.1.4; Principal Component Analysis (PCA): EIGENSOFT 6.1.4; ADMIXTURE clustering analysis: ADMIXTURE v1.2; Runs of homozygosity: hapROH v0.64; qpAdm modelling and F4-statistics: ADMIXTOOLS2 R package; f4-ratio: admixr R package; Phenotype predicton: HirisPlexS system. Results visualization and plot generation: R v. 4.2.3, ggplot2. Imputation: BCFtools v1.19; GLIMPSE v1.1.0.<br>Admixture dating: DATES.<br>Radiocarbon dates calibration and combination: Oxcal v4.4.4 and IntCal20.<br>Craniodental-based biological affinity: rASUDAS, CRANID: CR6bIND.<br>Facial reconstruction: Geomagic Freeform Plus 2024.0.87 software.

For manuscripts utilizing custom algorithms or software that are central to the research but not yet described in published literature, software must be made available to editors and reviewers. We strongly encourage code deposition in a community repository (e.g. GitHub). See the Nature Portfolio guidelines for submitting code & software for further information.

Data

Policy information about availability of data

All manuscripts must include a data availability statement. This statement should provide the following information, where applicable:

- Accession codes, unique identifiers, or web links for publicly available datasets
- A description of any restrictions on data availability
- For clinical datasets or third party data, please ensure that the statement adheres to our policy

Human reference genome build 37 (hs37d5) (https://ftp.1000genomes.ebi.ac.uk/vol1/ftp/technical/reference/phase2_reference_assembly_sequence/)
All the generated sequence data are available as bam files of aligned reads at the European Nucleotide Archive (ENA) under the project accession number PRJEB77356. Comparative ancient and modern genetic data were downloaded from the Allen Ancient DNA Resource (AADR, https://doi.org/10.7910/DVN/FFIDCW), the European Nucleotide Archive (ENA) under the accession number PRJEB59008 (Simoes et al. 2023), PRJEB50507 (Altınışık et al. 2022), and the European Genome-Phenome Archive (EGA) under the accession number EGAS00001000480 (Pagani et al. 2015; Egyptian low coverage). The origin of each genetic data is described in the Method section and in Supplementary Data Table 3.

Research involving human participants, their data, or biological material

Policy information about studies with human participants or human data. See also policy information about sex, gender (identity/presentation), and sexual orientation and race, ethnicity and racism.

Reporting on sex and gender	N/A
Reporting on race, ethnicity, or other socially relevant groupings	N/A
Population characteristics	N/A
Recruitment	N/A
Ethics oversight	N/A

Note that full information on the approval of the study protocol must also be provided in the manuscript.

Field-specific reporting

Please select the one below that is the best fit for your research. If you are not sure, read the appropriate sections before making your selection.

☒ Life sciences ☐ Behavioural & social sciences ☐ Ecological, evolutionary & environmental sciences

For a reference copy of the document with all sections, see nature.com/documents/nr-reporting-summary-flat.pdf

Life sciences study design

All studies must disclose on these points even when the disclosure is negative.

Sample size	No statistical methods were used to determine ancient DNA sample size a priori. Genomic, radiocarbon and isotope (13C, 15N, 18O, 88Sr/86Sr) data from an ancient individual from Egypt analysed in this study depend on the availability of human remains from ancient Egypt with preserved and retrievable ancient DNA sequences. This specimen is very rare, given the poor molecular preservation of human remains from this period in that region. Given the millions of genetic variants analysed, information about the genetic history can be retrieved.
Data exclusions	For the Nuwayrat genome and the comparative ancient genomes retrieved from bam files (from Altınışık et al. 2022 and Simoes et al. 2023), sequencing reads that did not map to the human reference hs37d5, were shorter than 35 bp, were library PCR amplification duplicates, had a mapping quality < 30 or contained indels, were removed from the bam files. Sequencing data with evidence of DNA contamination (SKO719A1706 and SKO719A1709) were also excluded. For single-stranded libraries (from the Nuwayrat genome), at C/T SNPs, forward mapping reads were discarded, and at G/A SNPs in reverse mapped reads were discarded using SequenceTools. For the double-stranded damage repair UDG-treated genomes (from Simoes et al. 2023), the first and last three bases of the sequenced reads were trimmed. For the double-stranded non-damage repaired genomes (from Altınışık et al. 2022), only transition sites were discarded. SNP calling was restricted to bases with base quality >30. We called pseudo-haploid SNPs, keeping only one random allele per individual at each position.<br>For the comparative present-day Egyptian genomes from Pagani et al. 2015, individuals with more than 10% missing genotypes, genotypes with missing call rates of 2% or failing the Hardy-Weinberg equilibrium test with p<1e-06 were discarded.<br>For all genomes, we kept only biallelic SNPs present in the Human Origin array or the 1240K capture SNP set.<br>In the whole comparative dataset, for pairs of first- and second-degree relatives, the individual with the lowest genomic coverage of the pair was excluded.<br>The PCA and ADMIXTURE clustering analyses were restricted to the transversions sites in the Human Origin array (111,208 SNPs). For the ADMIXTURE clustering analysis, all genomes (present-day and ancient) were pseudo-haploidised (we kept only on random allele at each diploid site per individual), genotypes were pruned for linkage disequilibrium resulting in 84,528 transversions sites being analysed. qpAdm and F4-statistics were restricted to the 1240K capture SNP set with CpG sites excluded, with only UDG-treated genomes and the Nuwayrat

used in the analyses.
F4-ratio to estimate Basal Eurasian ancestry restricted to the 1240K capture SNP set on transversion sites only.
Imputation was carried on the Nuwayrat genome and 200 ancient genomes from North Africa and West Asia associated with the Palaeolithic, Neolithic and Bronze Age, whole genome sequenced to >0.5X coverage or captured >2x coverage. For the genomes generated with UDG treatment, we first hard-trimmed the first and last three base pair of each reads and removed CpG sites and for the genome generate without UDG treatment, we removed all transition sites after SNP calling. We finally restricted the imputed genotypes to those with genotype probability GP ≥ 0.99 and minor allele frequency MAF ≥ 0.01, using the command bcftools filter -i 'MAX(FORMAT/GP)>=0.99 && INFO/RAF>=0.01&&INFO/RAF<=0.99' --set-GTs '.'.
Phenotype prediction and admixture dating were carried on this imputed dataset.

Replication	Several DNA extracts and multiple genomic libraries were generated for the newly reported sample from Nuwayrat, Egypt, and several rounds of sequencing were performed for each library (as reported in Supplementary Data Table S1), which acts as replication. Data was merged for downstream analysis after confirming similar results on contamination estimates and mitochondrial haplogroup. Thousands to millions of genetic markers were then analysed as an internal replication of the results. Detailed description of the methods used, including samples included in the dataset, software employed and respective parameters is available in the Method section.
Randomization	Randomization is not relevant to this study. Samples are grouped based on sampling locations, dates and genetic affinities.
Blinding	Blinding is not applicable for ancient specimens as the sampling locations and dates are known a-priori. In downstream data, analysis blinding is also not relevant since the newly generated ancient genome is co-analysed with previously published present-day and ancient human genomes.

Reporting for specific materials, systems and methods

We require information from authors about some types of materials, experimental systems and methods used in many studies. Here, indicate whether each material, system or method listed is relevant to your study. If you are not sure if a list item applies to your research, read the appropriate section before selecting a response.

Materials & experimental systems

n/a	Involved in the study	
☒	☐	Antibodies
☒	☐	Eukaryotic cell lines
☐	☒	Palaeontology and archaeology
☒	☐	Animals and other organisms
☒	☐	Clinical data
☒	☐	Dual use research of concern
☒	☐	Plants

Methods

n/a	Involved in the study	
☒	☐	ChIP-seq
☒	☐	Flow cytometry
☒	☐	MRI-based neuroimaging

Palaeontology and Archaeology

Specimen provenance	The specimen provenance is described in the Supplementary Information 1. The specimen was excavated at the Nuwayrat necropolis near Beni Hasan, Egypt. It was donated between 1902-04 by the Egyptian Antiquities Service to the members of the Beni Hasan excavation committee, and subsequently donated to the Institute of Archaeology, University of Liverpool, UK and exported under John Garstang export permit. The specimen was then given to the World Museum (previously Liverpool City Museum) in 1950
Specimen deposition	The World Museum of Liverpool (UK) is the sole curator of the specimen.
Dating methods	Three independent samples from the individual were directly radiocarbon dated using accelerator mass spectrometry (AMS) at the Beta Analytic Carbon dating laboratory (the two teeth that yielded ancient DNA) or at the Oxford Radiocarbon Accelerator Unit (femur), which date was obtained from the World Museum archive. Radiocarbon calibration was performed using OxCal v.4.4 and the IntCal20 dataset. The three dates were coherent and combined using OxCal v.4.4.

☒ Tick this box to confirm that the raw and calibrated dates are available in the paper or in Supplementary Information.

Ethics oversight	Permits for sampling and analyses of the archaeological material were obtained from the appropriate institutions.

Note that full information on the approval of the study protocol must also be provided in the manuscript.

Plants

Seed stocks	N/A
Novel plant genotypes	N/A
Authentication	N/A
